# Uptake of Ultrashort Chain, Emerging, and Legacy Per- and Polyfluoroalkyl Substances (PFAS) in Edible Mushrooms (*Agaricus spp*.) Grown in a Polluted Substrate

**DOI:** 10.1021/acs.jafc.2c03790

**Published:** 2023-03-08

**Authors:** Astrid Solvåg Nesse, Agnieszka Jasinska, Aasim Musa Ali, Oskar Sandblom, Trine A. Sogn, Jonathan P. Benskin

**Affiliations:** † Faculty of Environmental Sciences and Natural Resource Management, Norwegian University of Life Sciences, 1433 Ås, Norway; ‡ Lindum AS, 3036 Drammen, Norway; § Department of Vegetable Crops, Faculty of Horticulture, Poznan University of Life Sciences, 60-637 Poznań, Poland; ∥ Department of Contaminants and Biohazards, Institute of Marine Research, 5005 Bergen, Norway; ⊥ Faculty of Chemistry, Biotechnology and Food Science, Norwegian University of Life Sciences, 1433 Ås, Norway; # Department of Environmental Science, Stockholm University, 106 91 Stockholm, Sweden

**Keywords:** bioaccumulation factor, PFAS, fungi, mushroom, organic pollutants, biogas digestate, *Agaricus subrufescens*, *Agaricus bisporus*, circular economy

## Abstract

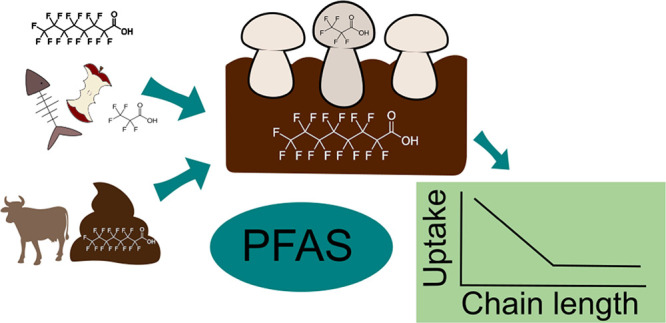

Uptake of 19 per- and polyfluoroalkyl substances (PFAS), including C3–C14 perfluoroalkyl carboxylic acids (PFCAs), C4, C6, and C8 perfluoroalkyl sulfonates (PFSAs), and four emerging PFAS, was investigated in two mushroom species (*Agaricus bisporus* and *Agaricus subrufescens*) cultivated in a biogas digestate-based substrate. Accumulation of PFAS in mushrooms was low and strongly chain-length dependent. Among the different PFCAs, bioaccumulation factors (log BAFs) decreased from a maximum of −0.3 for perfluoropropanoic acid (PFPrA; C3) to a minimum of −3.1 for perfluoroheptanoate (PFHpA; C7), with only minor changes from PFHpA to perfluorotridecanoate (PFTriDA; C13). For PFSAs, log BAFs decreased from perfluorobutane sulfonate (PFBS; −2.2) to perfluorooctane sulfonate (PFOS; −3.1) while mushroom uptake was not observed for the alternatives 3H-perfluoro-3-[(3-methoxy-propoxy)propanoic acid] (ADONA) and two chlorinated polyfluoro ether sulfonates. To the best of our knowledge, this is the first investigation of the uptake of emerging and ultra-short chain PFAS in mushrooms, and generally the results indicate very low accumulation of PFAS.

## Introduction

End-of-life material recycling is a critical step toward achieving a circular economy and ultimately reducing raw material demand and waste production, including greenhouse gas emissions.^1^ As an example, organic waste can be utilized for biogas production, a process in which methane is produced from the breakdown of organic matter by anaerobic microorganisms. Liquid and solid digestate generated by this process can be utilized as fertilizer in agronomic plant production in order to improve nutrient circularity.^2^ However, an important consideration when using digestates for agricultural fertilizer is the occurrence of micropollutants originating from the waste feedstocks which may persist during the digestion process. Occurrence of micropollutants in digestate-based fertilizer risks contamination of amended soil^3^ and accumulation in crops^4−6^ which can ultimately lead to human exposure, either by direct ingestion or leaching into groundwater and adjacent water bodies used for drinking water.^7^

One group of organic pollutants of high global concern which occur in organic wastes is the per- and polyfluoroalkyl substances (PFAS).^8−10^ PFAS encompass a diverse group of over 9000 substances that contain at least one perfluoromethyl (−CF_3_) or perfluoromethylene (−CF_2_−) group.^11^ The considerable strength of the C–F bond, combined with unique lipophobic and hydrophobic properties imparted by highly fluorinated aliphatic chains, has led to widespread use of PFAS in consumer products and industrial processes.^12^ The vast majority of PFAS are expected to persist in the environment or degrade to environmentally persistent end-products (i.e., perfluoroalkyl acids; PFAAs), the latter of which are highly mobile in water.^13,14^ Due to the risks associated with these substances, perfluorooctanesulfonic acid (PFOS), perfluorooctanoic acid (PFOA), perfluorohexanesulfonic acid (PFHxS), and long-chain perfluoroalkyl carboxylic acids (PFCAs; including related compounds) have been listed or proposed for listing as persistent organic pollutants (POPs) under the United Nations Stockholm Convention.^15,16^ Despite this initiative, alternative PFAS, for example with shorter perfluorinated chains or ether linkages within the fluorinated chain, continue to be produced.^17^ Short chain PFAS generally display reduced bioaccumulation potential in humans and wildlife but are equally as persistent as legacy PFAS.^14,18^ Moreover, uptake in crops has been shown to be largely dependent on sorption (either to soil, root-based lipids, or during the flow from leaves to fruit),^19,20^ resulting in greater accumulation of more hydrophilic/shorter chain length PFAS in plants.^5,19^

Mushrooms are a popular and nutritious food,^21^ which can be grown on a variety of organic waste products, including animal manure and biogas digestate.^22,23^ Due to their unique enzymatic machinery, mushrooms have received considerable attention as tools for bioremediation of different pollutants and agro-industrial wastes.^24^ Previous research investigating the uptake and degradation of contaminants by mushrooms has focused mostly on heavy metals^25−27^ and certain groups of organic contaminants such as polycyclic aromatic hydrocarbons^28,29^ and pesticides,^30^ but to the best of our knowledge, only a single study has investigated uptake of PFAS. In that work, Golovko et al.^31^ measured chain-length dependent uptake of 10 legacy PFAS in the edible oyster mushroom (*Pleurotus ostreatus*) using two different substrates (one with biogas digestate) containing 100 ng PFAS/g wet weight. Uptake was more efficient for perfluoroalkyl sulfonic acids (PFSAs) compared to PFCAs and was significantly reduced with increasing chain length. Moreover, uptake of PFAS was dependent on the PFAS concentration in the substrate, rather than the substrate composition itself.

In the current study, we build on the prior work of Golovko et al.^31^ by evaluating the uptake of 14 legacy, three emerging, and two ultrashort chain PFAS in two mushroom species (*Agaricus bisporus* and *Agaricus subrufescens*) cultivated in biogas digestate generated through an anaerobic digestion of food waste and manure to produce biogas. The objectives of this study were twofold: first, we sought to investigate the fate of ultra-short- and emerging replacement PFAS, for which there are a paucity of data. Second, we aimed to investigate inter-species differences in the uptake of PFAS in mushrooms. These data are the first to investigate uptake of emerging and ultra-short chain PFAS in mushrooms and provide new insights into the risks associated with using waste feedstocks for agricultural fertilizer.

## Materials and Methods

### Standards and Reagents

Authentic and isotopically labeled PFAS standards were purchased from Sigma, Wellington Laboratories, or Shanghai Syncia Co., Ltd. A full list of standards, including acronyms, is provided in Table S1. Methanol (HPLC-grade) was purchased from VWR. The water used throughout this work was either deionized, distilled, or grade 1 Milli-Q water, depending on the location of use (Stockholm University, NMBU, or Lindum).

### Dose Preparation

The mushroom substrate was spiked with 13 PFCAs (C2/trifluoroacetate [TFA] through C14/perfluorotetradecanoate [PFTeDA]), 3 PFSAs (PFBS, PFHxS, and PFOS), and the PFAA replacements ADONA (4.8-dioxa-3H-perfluorononaoic acid), F-53B (consisting of 9-chlorohexadecafluoro-3-oxanone-1-sulfonic acid [9Cl-PF3ONS] (major component) and 11-chloroeicosafluoro-3-oxaundecane-1-sulfonic acid [11Cl-PF3OUdS] (minor component)), and 2,3,3,3-tetrafluoro-2-(heptafluoropropoxy)propanoic acid [HFPO-DA/Gen-X] (Table S1). The doses of 6 mg of each PFCA and PFSA, 1.08 mg of F-53B, and 0.54 mg of ADONA, and Gen-X were dissolved in 100 mL of methanol at Stockholm University. For control, 100 mL of methanol was used. The two solutions were diluted to 2000 mL in grade 1 Milli-Q water at the Norwegian University of Life Sciences. Unfortunately, due to analytical challenges associated with TFA and Gen-X (the latter of which was due to degradation in acetonitrile^32^), results for these substances are not reported.

### Experimental Setup

**Figure 1:**
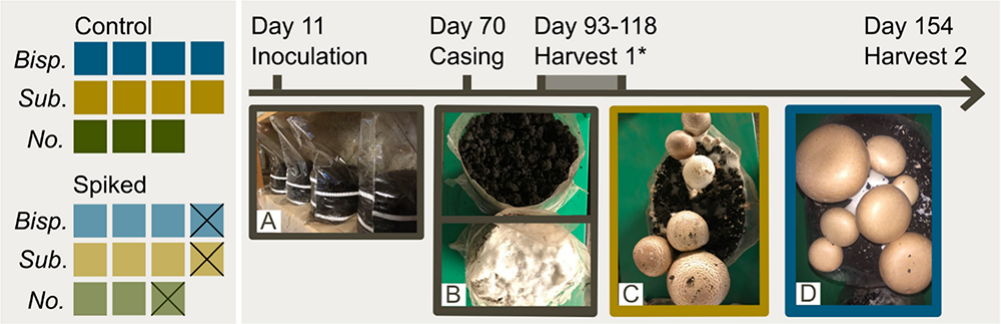
Mushroom growth. Twenty-two bags (i.e., experimental units) were filled with mushroom substrate, three of the spiked bags went rotten during the experiment (crossed out in the figure). (A) Bags filled with the substrate. (B) Mycelia has overgrown the substrate and casing is applied. (C) Emerging *A. subrufescens* pins, and mushroom hats. (D) Mature *A. bisporus* ready for harvest. *A few of the bags were harvested twice (i.e., harvests 1 and 2) during this period.

An overview of the experimental setup is provided in Figures 1 and S1. Briefly, two batches of mushroom substrate (“substrate”) were prepared, one control batch and one spiked with PFAS. Both control and spiked batches consisted of 11 subunits: four containing *A. subrufescens*, four with *A. bisporus*, and three without any mushrooms. Both whole mushrooms (i.e., the aboveground part of the mushroom, including the stem) and substrate were sampled at the same time for PFAS analysis, 1–2 times for each unit (depending on the number of “flushes” or “harvests,” i.e., the number of times the mushroom hats emerged from the substrate). Mushroom yield (g fresh weight) was also determined at each harvest (Table S2).

#### Preparation of the Mushroom Substrate

Each batch of substrate was prepared from 33 kg of biogas digestate mixed with control or spiking solution (digestate dry matter (DM): 4.3%), 10.5 kg of wheat straw, 300 g of chalk (Ca(OH)_2_), 300 g of gypsum (CaSO_4_), and 1 kg of activated garden compost for inoculation, to a total DM of 25%. The digestate was made from a feedstock of 73% household food waste and 27% manure and contained small amounts of native PFAS (see Results and Discussion). The substrate was composted (Phase I) for 9 days and pasteurized (Phase II) for 2 days (Figure S1). On days 0, 3, 6, 9, and 11, the substrate was mixed and sampled. On day 6, the control batch became too wet and about 1 L of liquid had gathered at the bottom of the drum. The liquid was removed as the compost would have become too wet if it was mixed into the compost again. This did not occur in the spiked compost. The preparation procedure is shown in Figure S1 along with temperature data for the two batches (mean of three temperature loggers in each batch). Further descriptions of the spiking procedure and equipment are found in the SI and Stoknes et al.^22^

#### Mushroom Cultivation

***Inoculation***. After pasteurization, each batch was split into 11 units. Four were inoculated with *A. subrufescens*, four with *A. bisporus*, and three were not inoculated (Figure 1). For inoculation, 90 g of granular spawn of the strains M7700 (*A. subrufescens*) and M7243 (*A. bisporus*) (Mycelia, Deinze, Belgium) were used (3% of the fresh substrate weight). Each unit was kept in a sealed 50 micron polypropylene bag of 7 L, with four linear ventilation filters (type PP50/SEU4/V40-51, SacO2 Microsac, Deinze, Belgium), at 25 °C (Figure 1A). ***Casing***. After the spawn overgrew the substrate (day 70, Figure 1B), the bags were opened and a 5–8 cm layer of casing (dark peat mixed with Ca(OH)_2_ and gypsum) was applied to initiate pinning (i.e., the emergence of fruiting bodies). The opened bags were moved to a cultivation chamber holding 25–30 °C and an air humidity of 70–75%. ***Pinning/fructification***. After 9 days, the mycelia had overgrown the surface of the casing and pinning (Figure 1) was initiated by moving *A. bisporus* bags to a chamber holding 17–18 °C. The tropical *A. subrufescens* and the no mushroom bags were kept in the first chamber, but the temperature was lowered to 20 °C for 5 days. The CO_2_ concentration was kept below 1000 ppm in both chambers. ***Harvesting***. The whole mushrooms were picked from the individual bags as they obtained maturity (Figure 1D), collecting all mushrooms from a single bag at once. After harvesting, the mushrooms again produce mushroom hats which were harvested the second time. Details of mushroom cultivation, sampling, and homogenization of samples are provided in the SI.

### Sample Characterization

Immediately after sampling, pH (determined using a ratio of 3:10 substrate: water, Milwaukee 802 pH/EC/DS meter), DM (105 °C, duplicate samples), and loss of ignition (550 °C) were determined. At harvests 1 and 2, pH was measured directly in the substrate with a “stick-in” pH meter (Table S3; G071505, BIOGRÓD) as it was not possible to sample enough material for standard pH analysis.

For analysis of PFAS, samples of substrate (oven dried) and mushrooms (fresh) were fortified with 10 ng of individual isotopically labeled internal standards (complete list provided in Tables S4 and S5) prior to extraction. Details of the extraction procedure are provided in the Supporting Information. Briefly, the substrate was extracted with methanol, while the mushrooms were extracted with acetonitrile. Both types of extracts were subjected to a dispersive carbon clean-up and then fortified with 10 ng of individual recovery standards and aqueous ammonium acetate prior to instrumental analysis. Two different instrumental methods were employed: PFPrA, PFBA, and PFPeA were analyzed by LC-high resolution mass spectrometry (HRMS) using a hybrid reversed phase/ion exchange column (Table S4).^33^ The remaining PFAS were analyzed by LC–MS/MS using a reversed phase column (Table S5). Further details on extraction, instrumental analysis, and quality control are provided in the SI, including results of matrix spike/recovery experiments involving both mushroom and fish muscle matrix (Tables S6 and S7) as well as analysis of NIST standard reference material (Table S8).

### Data Analysis

All statistics were performed in R Studio, version 4.1.2.^34^ Graphics were prepared in R Studio and Inkscape version 1.1.^35^ To assess differences in uptake between mushroom species, treatment (control/spiked), and harvest time for the spiked mushrooms, a mixed effect model was used. The experimental units were treated as random effects to avoid temporal autocorrelation. Due to the high number of nondetects in the mushroom samples for most compounds, the mixed effect model analysis was applied only for C8. Handling of data below the limit of quantification (LOQ) is described in detail in the SI.

For those replicates of the spiked treatment in which PFAS were detected, the bioaccumulation factors (BAFs) were calculated for each experimental unit at both harvests using the concentration in the mushroom divided by the concentration in the substrate (dry weight basis). For those replicates where the uptake was below the LOQ, worst case BAFs were calculated by dividing the mushroom-LOQ (calculated on dry weight basis) by the substrate concentration. Linear regression was used to determine the relationship between PFAS chain length and the logarithm of the BAFs. Assumptions were checked by inspection of the plots for “Residuals vs. Fitted” and “Normal Q–Q” (see SI for details).

## Results and Discussion

### Substrate Concentration

**Figure 2:**
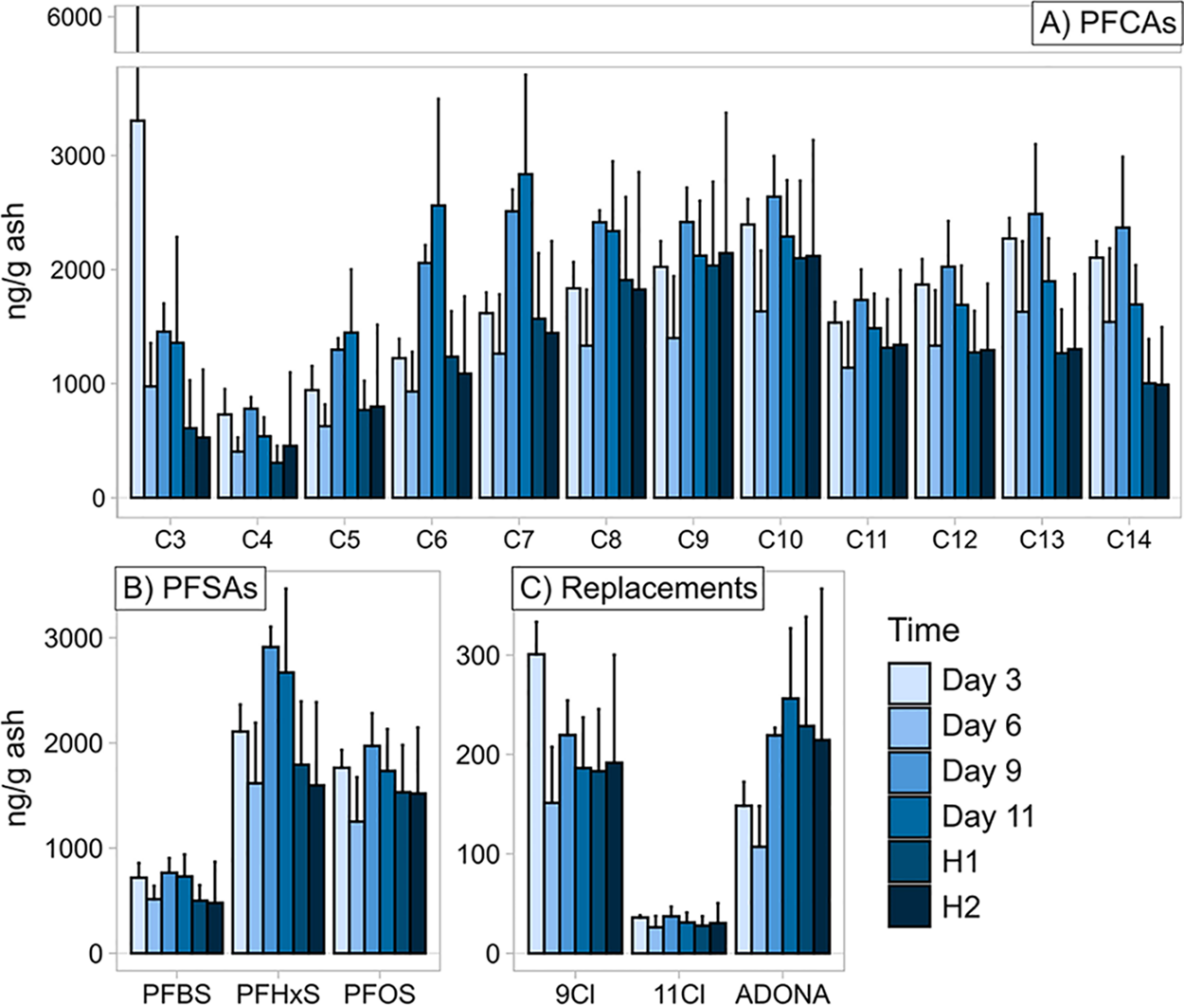
PFCAs (A), PFSAs (B), and replacements (“9Cl” = 9Cl-PF3ONS, “11Cl” = 11Cl-PF3OUdS) (C) in the spiked substrate (ng g^–1^ ash). Note that the y-axis has been cut due to the large standard deviations of C3 at day 3. H1 = Harvest 1, H2 = Harvest 2.

PFAS concentrations in substrates are reported on both a dry weight (Tables S9 and S10) and ash weight (Tables S11 and S12) basis. Ash-weight concentrations are generally considered more accurate due to the continuous degradation of the substrate, which would lead to an apparent upconcentration of PFAS with time for dry-weight concentrations. Analysis of PFAS in the spiked substrate revealed concentrations ranging from 18 to 83% of nominal (approximately 4000 ng g^–1^ ash for PFCAs and PFSAs) at day 3 (Figure 2). The possible occurrence of PFAA-precursors in the substrate (which, if present, could contribute to observed PFAA concentrations) was ruled out after observing that the PFAS concentrations in the control substrate were below 0.5% of that in the spiked substrate for all compounds at harvests 1 and 2. Removal of PFAS from the substrate via mushroom uptake was also limited based on the low concentrations observed in mushrooms (discussed further below).

### Mushroom Uptake of PFAS

PFAS concentrations determined in mushrooms are reported on both a fresh weight (Table S13) and dry weight (Table S14) basis. Overall, uptake of PFAS in both species of mushrooms on harvests 1 and 2 was limited with concentrations up to 14 ng g^–1^ dw in *A. subrufescens* and 28 ng g^–1^ dw in *A. bisporus* (for C3). Similar to observations in plants, uptake was strongly chain-length dependent with ultra-short chain PFAS displaying a much greater propensity for accumulation compared to long chain PFAS. PFAS concentrations in the mushrooms generally decreased from C3 to C7 before stabilizing at a concentration of 0.38–1.1 ng g^–1^ dw from C8 to C13 (no uptake of C12 and C14; Table 1). Note that C3, C4, C12, and C14, which were detected less frequently compared to the other PFAS, had the highest LOQs (Table S13). PFSA concentrations were in the same range as the long-chained PFCAs. A similar pattern was reported for uptake of C2–C6, C8, and PFOS in hydroponically grown wheat (*Triticum acstivnm* L.) where the concentration of C2 in the shoots was 13-fold higher than C3, which again was fourfold higher than for any of the other PFAS.^36^ Zhang et al.^36^ explained the considerable uptake of ultra-short chain PFAS by their high water solubility and small molecular size, leading to easier passage of the Casparian strip and translocation within the plant.

**Table 1:** Concentration (ng g^–1^ dw) and Total Uptake (ng) of PFAS in the Mushroom Hats Grown in the Spiked Substrate^a^

compound	concentration (ng g^–1^ dw)				total uptake (ng)			
	harvest 1		harvest 2		harvest 1		harvest 2	
	sub (*n* = 3)	bisp (*n* = 3)	sub (*n* = 2)	bisp (*n* = 2)	sub	bisp	sub	bisp
**C3**	4.2*	7.9*	14 (6.5)	28 (21)	74*	21*	95	117
**C4**	<LOQ	2.4*	<LOQ	9.0 (5.5)		6.3*		35
**C5**	<LOQ	0.46*	<LOQ	3.7 (4.4)		1.2*		18
**C6**	<LOQ	1.2 (1.1)	0.78 (0.12)	2.0 (1.2)		5.1	5.7	7.7
**C7**	<LOQ	0.91 (0.79)	0.50 (0.03)	0.47 (0.47)		4.1	3.7	2.1
**C8**	0.45 (0.11)	1.0 (0.55)	0.48 (0.04)	0.50 (0.15)	7.5	4.8	3.5	1.8
**C9**	<LOQ	0.80 (0.62)	<LOQ	0.43 (0.33)		3.7		1.8
**C10**	0.09*	0.79 (0.64)	0.34 (0.29)	0.60 (0.64)	1.7*	3.6	2.7	2.8
**C11**	<LOQ	0.71 (0.48)	0.38 (0.21)	0.59 (0.49)		3.3	3.0	2.5
**C13**	<LOQ	1.1 (0.94)	0.38 (0.21)	0.52 (0.39)		4.6	3.0	2.2
**PFBS**	<LOQ	0.77 (0.80)	0.46 (0.03)	1.2 (0.53)		3.0	3.4	4.5
**PFHxS**	0.09*	1.1 (0.83)	0.50 (0.38)	0.23 (0.13)	1.7*	5.3	4.0	0.9
**PFOS**	<LOQ	0.46 (0.28)	0.31 (0.25)	0.35 (0.30)		2.3	2.5	1.5

a Standard deviation is given in parenthesis. *There was uptake in only one replicate, the concentration/uptake divided on no. of replicates are given.

There was no observable uptake of F-53B components (9Cl-PF3ONS or 11Cl-PF3OUdS) or ADONA in any of the mushrooms perhaps due to the lower nominal concentration of these compounds (∼360 ng g^–1^ ash) compared to the PFAAs (∼4000 ng g^–1^ ash). Moreover, several plant uptake experiments have demonstrated that transport of F-53B from plant roots to shoots is limited with shoot concentrations less than 10% of those in roots (summarized by Zhang et al.^37^). However, in cattails, a frequently used bioremediation plant, the uptake of F-53B was higher.^38^ In both experiments performed by Zhang et al.,^37,38^ the shoot concentration of ADONA was several times higher than the F-53B concentration. Zhang et al.^37^ explained the difference by the fraction of water extractable compounds, which was about 2.3% for F-53B and 14% for ADONA in a soil with 29% organic carbon.

While the present work did not systematically investigate the influence of dose on PFAS uptake, some observations on the effect of dose could be made based on the occurrence of PFOA in control (i.e., unspiked) experiments. The concentration of C8 in spiked mushrooms, as estimated by a mixed effect model, was about twice as high as that in the control mushrooms (Table 2, *p* = 0.059). Considering that the level of C8 in the control substrate was below 0.5% of the concentration in the spiked substrate, the uptake of C8 by the control fungi was surprisingly high. C8 was found in nearly all replicates of both the control fungi and the spiked *A. subrufescens*, while other PFAS were taken up infrequently (Tables 1, 2, and S14). Furthermore, the level of C8 was similar to that of other PFAS in the control substrate, which makes it surprising that this particular compound should be taken up, while the other carboxylates generally were not.

**Table 2:** Linear Mixed Model Test on the Effect of Treatment (Control or Spiked), Time (Harvest 1 or 2), and Mushroom Species (*A. subrufescens* or *A. bisporus*) on the Concentration of PFOA (C8) in the Mushrooms (ng g^–1^ dw)^a^

	estimate	df	*p*-value
intercept	0.27 ± 0.13	10.27	0.056
treatment: spiked	0.32 ± 0.15	10.04	0.059
time: harvest 2	–0.04 ± 0.06	6.56	0.57
species: bisporus	0.22 ± 0.15	10.4	0.17

a The experimental units were included as a random factor. The intercept shows the estimate of the C8 concentration in control *A. subrufescens* mushrooms at harvest 1. The three bottom rows show the estimated additional effect of changing from control to spiked treatment, from harvest 1 to harvest 2, and from *A. subrufescens* to *A. bisporus*. df = degrees of freedom.

For the remaining PFAS, no statistical tests were performed as the number of observations was considered too low. However, inspection of the figures in Table 1 reveals that most PFAS were detected more often and (for some compounds) in higher concentrations in *A. bisporus* compared to *A. subrufescens*. PFAS were also taken up more frequently at harvest 2 compared to harvest 1. The uptake was 10–40-fold lower in both species from the present study compared to the oyster mushroom uptake assessed by Golovko et al.^31^ despite a higher spiking level (443 and 286 ng g^–1^ dw for each PFCA and PFSA, *A. spp*. and *P. ostreatus*, respectively). Different species of plants have also displayed differential PFAS accumulation with BAFs varying by up to eight orders of magnitude.^20^ Even different varieties of the same plant species may have variable uptake of PFAS.^39,40^

Experimental setup and growth conditions can influence the uptake of PFAS. From day 84 until the end of the experiment at day 154, *A. subrufescens* was maintained at 25 °C, while *A. bisporus* was cultivated at 17–18 °C. Since temperature influences the degradation rate of organic matter, the substrate composition for the two species may have become different, influencing the sorption and bioavailability of the target compounds. Likewise, the substrate of *P. ostreatus* was of a different composition from the *A. spp*. substrate; alder sawdust combined with either biogas digestate or wheat bran and calcium sulphate. The substrate preparation for the oyster mushroom experiment by Golovko et al.^31^ was shorter (8 h pasteurization), and the duration of the experiment was shorter as well, i.e., 28–25 days from inoculation to harvest (Golovko et al., personal communication).

### Bioaccumulation of PFAS in Mushrooms

**Figure 3:**
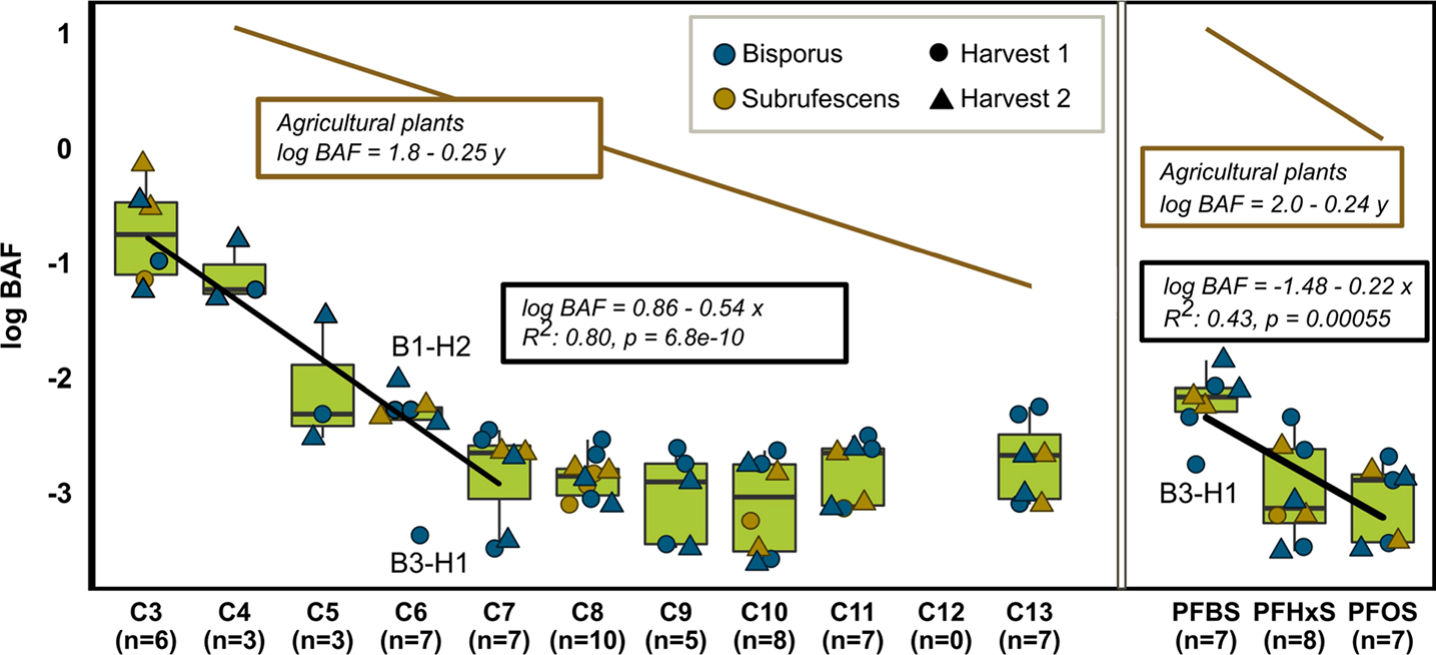
Bioaccumulation factors (BAFs) for PFAS in the mushroom hats, which were grown in spiked compost, and linear regression of log BAFs as a function of carbon chain length (*x*). Linear regression was based on combined data for both harvests (H1 and H2) and both species (S and B). There were a total of 10 datapoints for each PFAS (six for H1 and four for H2). When fewer datapoints are shown, it is because no uptake was detectable. Note that the nondetects are not included in the regression. Outliers are labeled: B1-H2: *A. bisporus*, replicate 1, harvest 2. B3-H1: *A. bisporus*, replicate 3, harvest 1. The regression lines for agricultural plants are taken from a review by Lesmeister et al.,^20^ where *y* is the number of perfluorinated carbons. Thus, *x* = *y* for the PFSAs and *x* = *y* + 1 for the PFCAs. The *p*-values are for the regression slopes.

The distinct chain-length dependency for C3 to C7 PFAS concentrations in mushrooms was reflected in calculated mean log BAFs, which decreased by 0.54 for each additional CF_2_-moiety among PFCAs (Figure 3; Table S15). From C7 to C13, the BAFs were almost equal. The PFSAs showed a similar pattern with a higher BAF for PFBS compared to PFHxS and PFOS. However, the median BAF was higher for PFOS than for PFHxS and linear regression explained only 43% of the variance (Figure 2). Similarly, Golovko et al. observed a similar mushroom concentration of PFHxS and PFOS, while the concentration of PFBS was about twice as high.^31^

The chain-length dependency can be explained by the bioavailability of PFAS since sorption of PFCAs and PFSAs generally increases with chain-length.^41−43^ In the study by Pereira et al.,^41^ less than 10% of the short chained PFAS such as C4, C5, and PFBS was sorbed in an organic soil layer (45% organic carbon), while an average of 99–100% of C10–C12, C14, and PFOS were sorbed. Similarly, Milinovic et al.^44^ assessed sorption to a peat soil (39% organic carbon) and found that up to 95–97% of PFOS, 70–81% of PFOA (C8), and 28–40% of PFBS were sorbed. In the study by Nguyen et al.,^43^ it was found that short-chained PFAAs such as C4–C6 PFCAs and C4–C5 PFSAs, as well as ADONA, were highly mobile in 10 different mineral soils (pH 6.2–7.7, 0.08–4.9% organic carbon), as seen by their negative log *K*_d_ values. These short-chained acids also appear to be less affected by changes in pH than the longer-chain length substances most probably because they already preferred the aqueous phase.^43^

The chain-length dependent uptake of PFAS has also been reported for oyster mushrooms and for agricultural plants. Golovko et al.^31^ found a decrease in oyster mushroom hat concentration of 1.68 and 5.4 ng g^–1^ dw for each additional CF_2_-moiety, for the PFCAs and PFSAs, respectively. In plants, a linear regression based on the median of 1800 BAFs for PFCAs and 500 for PFSAs showed a decrease in the BAFs from 0.24 to 0.25 log_10_ units for each additional CF_2_-moiety (Figure 3, Lesmeister et al.^20^). Different plant studies indicate that the chain-length dependent uptake of PFAS arises not only due to the bioavailability in the growth medium but also from a selective transport within the plant. Even in hydroponic studies, where sorption does not restrict uptake, shoot-BAFs show a chain-length dependency.^20,45^ The transport mechanism of polar organic chemicals such as PFAS in fungi is, however, unknown. Available literature on mechanisms of chemical uptake in fungi has mainly focused on metals and nutrients (e.g., iron^46,47^), textile dyes,^48,49^ and hydrophobic organic compounds connected to oil spills and fuel, such as alkanes and PAHs (e.g.,^50−53^).

The higher accumulation of PFCAs compared to PFSAs with equal number of CF_2_-moieties, which is often observed in plants (e.g.,^54^), was not seen in the present study. For example, in the spiked *A. bisporus*, at both harvests, the log BAFs differ by only 0.5–9% when comparing the pairs PFBS/C5, PFHxS/C7, and PFOS/C9 (for the same species, time, and treatment).

Only PFBS in one experimental unit had a BAF above 1 (log BAF above 0), which may indicate a potential for bioaccumulation in a scientific context as the concentration is higher in the mushroom compared to the growing media.^55^ Regulatory criteria are, however, set higher. For example, under REACH,^56^ substances must have a bioconcentration factor (BCF) in aquatic species of at least 2000 to be classified as bioaccumulative and 5000 to be considered very bioaccumulative, corresponding to log BCFs of 3.3 and 3.7, respectively. On the basis of the data provided by Golovko et al.,^31^ approximate BAFs were calculated also for oyster mushrooms (samples of mushrooms and substrate were not taken at the same day). Similar to what has been observed in the present study, the BAFs for oyster mushrooms were essentially equal for C7 to C12 (−1.3 to −1.4), and slightly higher for C6 (−0.96). A similar pattern was observed for PFSAs in oyster mushrooms; a higher BAF for PFBS (−0.72) and similar BAFs for PFHxS (−1.4) and PFOS (−1.5). Although the BAFs calculated based on data for the oyster mushrooms were higher than the BAFs calculated in the present study, the oyster mushroom log BAFs were also well below 0.

Since there were a high number of nondetects for PFAS in the mushrooms, worst-case BAFs were calculated to assess a theoretical potential for uptake of PFAS in the mushrooms based on the LOQs (Table S16). The worst-case BAFs were all well below 1 for compounds in the spiked treatment and for about half of the compounds in the control treatment. The worst-case BAFs were particularly high for those compounds having a relatively higher mushroom LOQs (e.g., up to 27 for C14) and for those having a low substrate concentration (e.g., up to 14 for 11Cl). Nevertheless, compared to the BCF regulation limit of 2000, the potential for uptake of PFAS in mushrooms is clearly very low.

The spiking concentrations in the present experiment were in the same order of magnitude as concentrations reported in French urban wastes such as sewage sludge and municipal waste (average sum of 160 PFAS was 307 ng g^–1^ dm, and the median was 265 ng g^–1^ dm).^9^ Overall, the limited uptake of PFAS into the edible parts of the fungi suggests that it is possible to use PFAS-containing waste material to produce mushrooms that are safe for human consumption. However, considering that PFAS are one of many organic contaminants which may occur in sewage sludge, additional mushroom uptake studies are urgently needed. Of particular importance are pharmaceuticals and personal care products, which occur widely in sewage sludge. Investigations into the uptake of these substances in mushrooms by our lab are ongoing and will be presented in a companion paper to the present study in the future.

## Supporting Information Available

Additional information on target analytes, methods (incl. instrumental parameters for UPLC/MS/MS and quality control), PFAS concentrations in the substrate and mushrooms, mushroom yield, pH, BAFs, and statistical analysis (PDF)
