# Supplementary material for: Uptake of Ultrashort Chain, Emerging, and Legacy Per- and Polyfluoroalkyl Substances (PFAS) in Edible Mushrooms (Agaricus spp.) Grown in a Polluted Substrate
Source: J Agric Food Chem. 2023 Mar 8;71(11):4458–65. doi: 10.1021/acs.jafc.2c03790 (PMC10037327; doi:10.1021/acs.jafc.2c03790)
Supplement: Supplementary file 1 — jf2c03790_si_001.pdf [file jf2c03790_si_001.pdf]

Supplementary Information

Uptake of ultrashort chain, emerging, and legacy per- and polyfluoroalkyl substances (PFAS) in edible mushrooms (*Agaricus spp.*) grown in a polluted substrate

Astrid Solvåg Nesse*[1], Agnieszka Jasinska[2,3], Aasim Musa Ali[4,5], Oskar Sandblom[6], Trine Aulstad Sogn[1], Jonathan P. Benskin[6]

[1] Faculty of Environmental Sciences and Natural Resource Management, Norwegian University of Life Sciences, 1433 Ås, Norway
[2] Lindum AS, 3036 Drammen, Norway
[3] Department of Vegetable Crops, Faculty of Horticulture, Poznan University of Life Sciences, 60-637 Poznań, Poland
[4] Department of Contaminants and Biohazards, Institute of Marine Research, 5005 Bergen, Norway
[5] Faculty of Chemistry, Biotechnology and Food Science, Norwegian University of Life Sciences, 1433 Ås, Norway
[6] Department of Environmental Science, Stockholm University, 106 91 Stockholm, Sweden
* astrid.solvag.nesse@nmbu.no, phone: 00 47 67 23 18 99

Contents

Spiking procedure p. S19
Preparation of mushroom substrate p. S19
Sampling procedure for the mushroom substrate p. S20
Temperature logging p. S21
Substrate extraction procedure p. S21
Mushroom extraction procedure p. S21
Instrumental analysis p. S22
Quality control p. S22
Substitution of values below the limit of quantification p. S23
Results of statistical analysis p. S24
References p. S27

List of tables

S1. List of PFAS standards p. S3
S2. Mushroom yield p. S4
S3. pH in the substrate p. S5
S4. Instrumental parameters for PFPrA, PFBA, PFPeA p. S6
S5. Target compounds and internal standards, selected instrumental parameters for quantification by UPLC/MS/MS p. S7
S6. Spike/recovery in mushrooms p. S8
S7. Spike/recovery in fish muscles p. S9
S8. NIST SRM 2781 in sludge p. S10
S9. PFAS in control substrate, dry weight basis, and substrate LOQs p. S11
S10. PFAS in spiked substrate, dry weight basis p. S12
S11. PFAS in control substrate, ash basis p. S13
S12. PFAS in spiked substrate, ash basis p. S14
S13. PFAS in mushrooms, fresh weight, and mushroom LOQs p. S15
S14. PFAS in mushrooms, dry weight p. S16
S15. Log10 Bioaccumulation factors p. S17
S16. "Worst-case"- Bioaccumulation factors p. S18
S17. Decision rule for imputation of observations below the limit of quantification (LOQ) p. S23

List of figures

Figure S1. Preparation of mushroom substrate p. S19
Figure S2. Result of mixed effect model for PFOA p. S24
Figure S3. Result of linear regression on PFCA BAFs p. S25
Figure S4. Diagnostic plot for linear regression on PFCA BAFs p. S25
Figure S5. Result of linear regression on PFSA BAFs p. S26
Figure S6. Diagnostic plot for linear regression on PFSA BAFs p. S26

Table S1. List of PFAS standards used for mushroom dosing experiment along with abbreviations and CAS-number

Full name		Acronym		CAS-number
Trifluoroacetic acid		TFA	C2	76-05-1
Perfluoropropanoic acid		PFPrA	C3	422-64-0
Perfluorobutanoic acid		PFBA	C4	375-22-4
Perfluoropentanoic acid		PFPeA	C5	2706-90-3
Perfluorohexanoic acid		PFHxA	C6	307-24-4
Perfluoroheptanoic acid		PFHpA	C7	375-85-9
Perfluorooctanoic acid		PFOA	C8	335-67-1
Perfluorononanoic acid		PFNA	C9	375-95-1
Perfluorodecanoic acid		PFDA	C10	335-76-2
Perfluoroundecanoic acid		PFUnDA	C11	2058-94-8
Perfluorododecanoic acid		PFDoDA	C12	307-55-1
Perfluorotridecanoic acid		PFTriDA	C13	72629-94-8
Perfluorotetradecanoic acid		PFTeDA	C14	376-06-7
Perfluorobutane sulfonic acid		PFBS		375-73-5
Perfluorohexane sulfonic acid		PFHxS		355-46-4
Perfluorooctane sulfonic acid		PFOS		1763-23-1
4.8-dioxa-3H-perfluorononaoic acid		ADONA		958445-44-8
2,3,3,3-tetrafluoro-2-(heptafluoropropoxy)propanoic acid		Gen-X		13252-13-6
F-53B	9-chlorohexadecafluoro-3-oxanone-1-sulfonic acid (major component)	9Cl-PF3ONS		73606-19-6
	11-chloroeicosafluoro-3-oxaundecane-1-sulfonic acid (minor component)	11Cl-PF3OUdS		83329-89-9

Table S2. Mushroom yield (g fresh weight), with standard deviation in parenthesis. Number of replicates was four for the control treatment, three for the spiked treatment at harvest 1 (H1), and two for the spiked treatment at harvest 2 (H2).

	Agaricus subrufescens		*Agaricus bisporus*	
	H1	H2	H1	H2
Control	114 (56)	68 (15)	154 (22)	42 (21)
Spiked	127 (22)	45 (7)	52 (33)	26 (27)

Table S3. pH of the substrate, with standard deviation in parenthesis. C = control, S = spiked

	Control			Spiked		
Digestate	7.9					
Day 3	9.0 (0.01)			9.0 (0.05)		
Day 6	8.8 (0.06)			8.9 (0.1)		
Day 9	9.2 (0.06)			9.1 (0.2)		
	Sub - C	Bisp - C	No - C	Sub - S	Bisp - S	No - S
Inoculation	9.0 (0.08)	8.8 (0.2)	9.1 (0.09)	9.0 (0.1)	8.9 (0.04)	9.0 (0.2)
Casing	6.2 (0.4)	6.7 (0.1)	8.0 (0.3)	6.4 (0.3)	6.9 (0.7)	7.7 (0.4)
Harvest 1	5.6 (0.06)	7.6 (0.7)	8.7 (0.09)	5.8 (0.3)	8.3 (0.6)	8.5 (0.1)
Harvest 2	5.9 (0.4)	8.4 (0.2)	8.2 (0.03)	7.1 (0.9)	8.4 (0.06)	8.1 (0.02)

Table S4. Instrumental parameters for PFPrA. PFBA. and PFPeA

**Instrument**	Dionex Ultimate 3000 liquid chromatograph coupled to a Q Exactive HF Orbitrap (Thermo Scientific)		
**Column**	Thermo Scientific Acclaim Trinity P1 Charged nanopolymer silica hybrid (3µm. 2.1 × 100mm)		
**Mobile Phase**	A: 20 mM ammonium acetate in SPE-polished HPLC grade water<br>B: 20 mM ammonium acetate in methanol		
**Injection volume**	10 µL		
**Column temperature**	40 ˚C		
**Flow rate**	0.2 ml/min		
**Gradient**	**Time (min)**	**%A**	**%B**
	0	75	25
	0.25	75	25
	10	5	95
	12	5	95
	13	75	25
	16	75	25
Spray voltage	3.7 kV		
Capillary Temp	320 C		
Sheath gas	30		
Aux gas	10		
Aux gas heater	320 C		
S-lens RF level	50		
polarity	Negative		
Resolution	120 000		
AGC target	3e6		
Maximum IT	250 ms		
Scan range	100 – 1500 m/z		
resolution	15 000		
AGC target	2e5		
Maximum IT	30 ms		
Loop count	5		
Isolation window	0.4 m/z		
Collision energy	35		
**Inclusion list**			
**Target**	**Exact mass (m/z)**	**Internal standard**	**Exact mass (m/z)**
**PFPrA**	162.98239	$^{13}C_4$PFBA	216.99262
**PFBA**	212.97920	$^{13}C_4$PFBA	216.99262
**PFPeA**	262.97601	$^{13}C_5$PFPeA	267.99278

Table S5. Target compounds and internal standards, selected instrumental parameters for quantification by UPLC/MS/MS.

**Instrument**	Acquity UPLC + Xevo TQ-S (Waters Corp. Milford. MA)			
**Column**	Guard: BEH C18 (5 × 2.1 mm. 1.7 µm)<br>Analytical: BEH C18 (50 × 2.1 mm. 1.7 µm)			
**Mobile Phase**	A: 90 % water and 10 % acetonitrile containing 2 mM ammonium acetate.<br>B: 100 % acetonitrile containing 2 mM ammonium acetate.			
**Injection volume**	5 µL			
**Column temperature**	40 ˚C			
**Gradient**	**Time (min)**	**%A**	**%B**	**Flow Rate (ml/min)**
	0.0	90	10	0.40
	0.5	90	10	0.40
	5.0	20	80	0.40
	5.1	0	100	0.40
	6.6	0	100	0.40
	8.0	0	100	0.55
	10.0	90	10	0.40
**Source temperature**	150 ˚C			
**Desolvation temperature**	350 ˚C			
**Cone gas flow rate**	150 l/hr (nitrogen)			
**Desolvation gas**	650 l/hr (nitrogen)			
**Nebulizer gas**	7 bar			
**Capillary voltage**	3000 V			

**Abbreviation**[¤]	**Precursor Ion**	**Quantitative Product ion**	**Qualitative product ion**	**Internal standard**	**IS transition**
PFHxA	313	269	119	$^{13}C_2$-PFHxA	315>270
PFHpA	363	319	169	$^{13}C_4$-PFHpA	367>322
PFOA	413	169	369	$^{13}C_4$-PFOA	417>372
PFNA	463	419	219	$^{13}C_5$-PFNA	468>423
PFDA	513	469	269	$^{13}C_2$-PFDA	515>470
PFUnDA	563	519	269	$^{13}C_2$-PFUnDA	565>520
PFDoDA	613	569	169	$^{13}C_2$-PFDoDA	615>570
PFTrDA	663	619	169	$^{13}C_2$-PFDoDA	615>570
PFTeDA	713	669	169	$^{13}C_2$-PFDoDA	615>570
ADONA	377	251	85	$^{13}C_4$-PFOA	417>372
9Cl-PF3ONS	531	351	83	$^{13}C_4$-PFOS	503>80
11Cl-PF3OUdS	631	451	83	$^{13}C_4$-PFOS	503>80
PFBS	299	80	99	$^{18}O_2$-PFHxS	403>84
PFHxS	399	80	99	$^{18}O_2$-PFHxS	403>84
PFOS	499	80	99	$^{13}C_4$-PFOS	503>80
PFDS	599	80	99	$^{13}C_4$-PFOS	503>80
				$^{13}C_8$-PFOS (recovery standard)	507>80
				$^{13}C_8$-PFOA (recovery standard)	421>376

Table S6. Results of spike/recovery experiment performed in mushrooms (n=6)

		PFPrA	PFBA	PFPeA	PFHxA	PFHpA	PFOA	PFNA	PFDA	PFUnDA	PFDoDA	PFTriDA	PFTeDA	PFBS	PFHxS	PFOS	9Cl-PF3ONS	11Cl-PF3OUdS	ADONA
**Method**		**P1**	**P1**	**P1**	**C18**	**C18**	**C18**	**C18**	**C18**	**C18**	**C18**	**C18**	**C18**	**C18**	**C18**	**C18**	**C18**	**C18**	**C18**
**Spiking amount (ng)**		50	39	39	39	39	39	39	39	39	39	39	39	39	39	39	39	39	39
% recovery	1	106	103	121	113	107	107	106	111	115	117	112	111	92	103	109	118	92	89
	2	93	96	116	113	109	102	108	117	118	116	107	111	84	99	108	102	78	86
	3	143	97	112	106	107	106	98	112	121	112	113	111	94	98	107	108	92	101
	4	98	102	110	106	107	100	99	105	113	126	113	107	89	99	106	106	83	101
	5	111	102	125	104	108	106	107	112	118	123	101	95	89	101	102	108	84	89
	6	118	99	118	109	106	104	104	116	120	118	111	128	84	95	104	116	100	88
**AVG**		**112**	**100**	**117**	**108**	**107**	**104**	**104**	**112**	**118**	**119**	**110**	**110**	**89**	**99**	**106**	**110**	**88**	**92**
**STDEV**		18	3	5	4	1	3	4	4	3	5	5	10	4	3	3	6	8	7

Table S7. Results of spike/recovery experiment performed in fish muscle (n=6)

		PFPrA	PFBA	PFPeA	PFHxA	PFHpA	PFOA	PFNA	PFDA	PFUnDA	PFDoDA	PFTriDA	PFTeDA	PFBS	PFHxS	PFOS	9Cl-PF3ONS	11Cl-PF3OUdS	ADONA
**Method**		**P1**	**P1**	**P1**	**C18**	**C18**	**C18**	**C18**	**C18**	**C18**	**C18**	**C18**	**C18**	**C18**	**C18**	**C18**	**C18**	**C18**	**C18**
**Spiking amount (ng)**		40	2.5	2.5	2.5	2.5	2.5	2.5	2.5	2.5	2.5	2.5	2.5	2.5	2.5	2.5	2.5	2.5	2.5
% recovery	1	108	107	104	154	118	133	131	128	121	138	117	127	156	123	131	111	49	138
	2	108	110	117	136	113	118	127	133	107	128	101	119	183	123	137	131	67	117
	3	106	106	99	126	115	115	110	127	109	105	92	101	164	121	120	98	46	112
	4	100	114	95	127	108	121	116	121	158	105	98	109	159	116	126	95	45	112
	5	109	115	119	132	119	126	136	117	125	134	112	116	200	150	133	121	51	108
	6	95	102	91	133	107	113	117	115	136	131	85	103	145	86	128	105	47	113
**AVG**		**104**	**109**	**104**	**135**	**113**	**121**	**123**	**124**	**126**	**124**	**101**	**112**	**168**	**120**	**129**	**110**	**51**	**117**
**STDEV**		**5**	**5**	**12**	**10**	**5**	**7**	**10**	**7**	**19**	**15**	**12**	**10**	**20**	**20**	**6**	**14**	**8**	**11**

Table S8. Results of analysis of NIST SRM 2781 in sludge. Reference values for 9Cl-PF3ONS, 11Cl-PF3OUdS, ADONA, and PFPrA are unavailable.

	PFBA	PFPeA	PFHxA	PFHpA	PFOA (L+Br)	PFNA	PFDA	PFUnDA	PFDoDA	PFTriDA	PFTeDA	PFBS	PFHxS	PFOS (L+Br)[a]
**Method**	**P1**	**P1**	**C18**	**C18**	**C18**	**C18**	**C18**	**C18**	**C18**	**C18**	**C18**	**C18**	**C18**	**C18**
1	2.13	2.65	6.62	4.38	12.7	1.67	3.07	<LOQ	0.99	0.46	<LOQ	<LOQ	4.48	168
2	2.38	2.85	7.75	4.77	16.1	1.64	3.60	<LOQ	1.23	0.23	<LOQ	<LOQ	7.03	203
3	2.49	3.00	9.03	4.68	17.5	1.82	3.31	2.00	1.21	0.38	<LOQ	<LOQ	7.54	241
**AVG**	**2.33**	**2.84**	**7.80**	**4.61**	**15.4**	**1.71**	**3.33**	**<2.00**	**1.14**	**0.36**	**<0.04**	**<0.03**	**6.35**	**204**
**STDEV**	0.19	0.17	1.21	0.21	2.46	0.10	0.27	-	0.14	0.11	-	-	1.64	36
**Range of values reported[b,c]**	3.34-35.9	<3-9.56	9.97-17	4.83-9.6	18.7-31	1.7-6.59	2.67-6.05	<1.5-7.13	<1.5-4.81	<1.4-24	<0.63-1	<0.186-34.9	5-10	166-373

[a]sum of linear and branched isomers acquired from the m/z 499/80 transition. [b] Munoz et al. (2021). [c] Reiner et al. (2015)

Table S9. Limit of quantification (LOQ) and PFAS levels in the control substrate [ng g^{-1} dw] with standard deviation in parenthesis. At day 0, only the levels in the digestate were measured (not the complete compost mix). *Only one out of three replicates had levels above the LOQ. Mean is given as the level in that sample divided on the number of replicates. See Table S17 for decision rule on when to substitute observations below the LOQ. On Day 0, the levels of PFAS were measured in the digestate, not in the compost mixture.

						Inoculation			Harvest 1			Harvest 2		
	**LOQ**	**Day 0**	**Day 3**	**Day 6**	**Day 9**	**Sub**	**Bisp**	**No**	**Sub**	**Bisp**	**No**	**Sub**	**Bisp**	**No**
C3	2.40	<LOQ	4.0 (2.1)	1.5*	7.1 (1.1)	12 (4.7)	5.4 (3.1)	5.6 (2.8)	<LOQ	<LOQ	<LOQ	<LOQ	<LOQ	<LOQ
C4	1.06	<LOQ	<LOQ	<LOQ	<LOQ	0.47*	<LOQ	<LOQ	<LOQ	<LOQ	<LOQ	<LOQ	<LOQ	<LOQ
C5	0.25	<LOQ	<LOQ	<LOQ	0.07*	0.49 (0.31)	0.25 (0.09)	0.41 (0.17)	3.1 (1.7)	0.53 (0.56)	0.50 (0.24)	1.4 (0.43)	0.59 (0.11)	1.1 (0.25)
C6	0.04	<LOQ	0.22 (0.17)	0.72 (0.38)	0.68 (0.12)	1.6 (0.53)	0.81 (0.30)	1.1 (0.28)	1.4 (0.47)	1.6 (0.83)	1.0 (0.29)	1.3 (0.11)	1.6 (0.11)	2.3 (0.45)
C7	0.04	<LOQ	0.16 (0.14)	0.47 (0.35)	0.77 (0.06)	1.1 (0.51)	0.57 (0.36)	0.96 (0.32)	0.83 (0.29)	0.99 (0.83)	0.62 (0.27)	0.90 (0.11)	1.1 (0.14)	1.8 (0.46)
C8	0.04	0.29 (0.08)	0.47 (0.11)	0.67 (0.34)	0.84 (0.08)	1.1 (0.50)	0.72 (0.20)	0.84 (0.12)	1.33 (0.39)	1.4 (0.78)	0.57 (0.22)	1.3 (0.14)	1.3 (0.11)	1.6 (0.28)
C9	0.04	0.08 (0.04)	0.35 (0.14)	0.77 (0.46)	0.64 (0.28)	0.66 (0.40)	0.50 (0.20)	0.52 (0.16)	0.80 (0.30)	1.2 (0.75)	0.31 (0.20)	1.1 (0.20)	1.1 (0.20)	0.93 (0.18)
C10	0.03	0.17 (0.03)	0.48 (0.20)	0.87 (0.44)	0.80 (0.38)	0.74 (0.42)	0.61 (0.31)	0.54 (0.07)	0.94 (0.34)	1.4 (0.77)	0.45 (0.28)	1.3 (0.25)	1.4 (0.32)	1.0 (0.20)
C11	0.04	0.13 (0.05)	0.29 (0.08)	0.49 (0.59)	0.51 (0.18)	0.33 (0.19)	0.36 (0.19)	0.25 (0.09)	0.47 (0.14)	0.93 (0.44)	0.35 (0.19)	0.74 (0.26)	0.78 (0.22)	0.56 (0.15)
C12	0.03	0.06 (0.04)	0.42 (0.23)	0.89 (0.27)	0.51 (0.24)	0.39 (0.26)	0.42 (0.22)	0.28 (0.10)	0.44 (0.16)	1.3 (0.73)	0.35 (0.15)	0.67 (0.27)	0.78 (0.32)	0.56 (0.23)
C13	0.07	0.13 (0.07)	0.51 (0.28)	1.2 (0.24)	0.60 (0.19)	0.37 (0.25)	0.46 (0.19)	0.32 (0.19)	0.41 (0.16)	1.5 (0.95)	0.37 (0.14)	0.63 (0.25)	0.77 (0.38)	0.56 (0.17)
C14	0.03	0.03*	0.44 (0.24)	1.1 (0.15)	0.58 (0.12)	0.37 (0.26)	0.40 (0.27)	0.28 (0.04)	0.32 (0.14)	1.6 (1.64)	0.28 (0.11)	0.51 (0.20)	0.60 (0.29)	0.53 (0.21)
PFBS	0.03	<LOQ	<LOQ	0.21 (0.19)	0.05*	0.20 (0.13)	0.12 (0.12)	0.23 (0.07)	0.33 (0.09)	0.37 (0.19)	0.23 (0.05)	0.23 (0.04)	0.26 (0.08)	0.44 (0.18)
PFHxS	0.04	0.30*	0.55 (0.29)	0.52 (0.20)	0.54 (0.30)	0.87 (0.40)	0.59 (0.20)	0.75 (0.03)	0.76 (0.27)	0.90 (0.59)	0.12 (0.06)	0.70 (0.18)	0.59 (0.10)	0.44 (0.23)
PFOS	0.05	0.82 (0.47)	7.5 (5.7)	2.1 (2.7)	3.3 (2.6)	13 (7.6)	7.5 (5.7)	5.8 (3.0)	0.65 (0.19)	0.84 (0.35)	0.09 (0.07)	0.76 (0.20)	0.67 (0.15)	0.24 (0.11)
9Cl	0.03	<LOQ	0.05 (0.02)	0.11 (0.03)	<LOQ	<LOQ	<LOQ	<LOQ	<LOQ	0.08 (0.05)	<LOQ	0.08 (0.04)	0.07 (0.01)	0.01*
11Cl	0.04	<LOQ	<LOQ	<LOQ	<LOQ	<LOQ	<LOQ	<LOQ	<LOQ	<LOQ	<LOQ	<LOQ	<LOQ	<LOQ
ADONA	0.04	<LOQ	<LOQ	0.02*	<LOQ	0.06 (0.03)	0.04 (0.01)	<LOQ	0.05 (0.02)	0.05*	0.05 (0.02)	0.07 (0.02)	0.09 (0.04)	0.15 (0.05)

Table S10. Limit of quantification (LOQ) and PFAS levels in the spiked substrate [ng g^{-1} dw], with standard deviation in parenthesis.

					Inoculation			Harvest 1			Harvest 2		
	LOQ	Day 3	Day 6	Day 9	Sub	Bisp	No	Sub	Bisp	No	Sub	Bisp	No
C3	2.40	265 (95)	158 (88)	203 (49)	100 (46)	142 (16)	144 (57)	91 (75)	123 (94)	178 (93)	52 (43)	144 (72)	184 (114)
C4	1.06	96 (32)	64 (30)	107 (4.9)	72 (27)	106 (10)	79 (26)	77 (52)	77 (43)	102 (39)	49 (38)	78 (27)	186 (135)
C5	0.25	124 (31)	99 (41)	179 (11)	191 (92)	305 (15)	184 (62)	189 (56)	214 (94)	226 (53)	131 (82)	169 (42)	293 (67)
C6	0.04	161 (28)	146 (66)	286 (41)	332 (144)	536 (23)	341 (132)	282 (29)	348 (145)	386 (67)	216 (112)	263 (34)	366 (31)
C7	0.04	213 (31)	198 (90)	349 (51)	367 (119)	570 (14)	418 (162)	330 (33)	459 (199)	503 (94)	304 (140)	358 (46)	465 (59)
C8	0.04	242 (39)	210 (92)	334 (17)	307 (85)	455 (15)	361 (99)	401 (24)	563 (249)	605 (171)	378 (137)	448 (66)	598 (70)
C9	0.04	267 (41)	217 (87)	333 (17)	281 (71)	403 (9.1)	342 (64)	452 (26)	590 (268)	629 (174)	442 (149)	516 (78)	717 (97)
C10	0.03	315 (42)	254 (86)	363 (30)	308 (83)	424 (8.7)	378 (55)	496 (29)	598 (259)	625 (154)	465 (123)	561 (65)	636 (66)
C11	0.04	202 (32)	176 (61)	238 (23)	205 (53)	270 (8.8)	246 (36)	317 (15)	368 (163)	392 (119)	304 (82)	336 (42)	410 (50)
C12	0.03	246 (42)	205 (67)	277 (33)	229 (62)	307 (14)	286 (30)	320 (12)	329 (129)	403 (117)	297 (62)	331 (51)	394 (76)
C13	0.07	299 (39)	250 (83)	340 (57)	255 (65)	346 (21)	324 (24)	322 (7.0)	300 (120)	440 (113)	301 (63)	308 (65)	415 (69)
C14	0.03	277 (31)	235 (83)	323 (64)	220 (56)	301 (24)	314 (36)	257 (6.3)	206 (76)	394 (144)	230 (43)	225 (46)	328 (78)
PFBS	0.03	95 (22)	80 (23)	105 (10)	102 (34)	144 (4.1)	100 (16)	135 (28)	141 (59)	129 (31)	98 (41)	102 (15)	156 (54)
PFHxS	0.04	278 (48)	253 (103)	402 (17)	350 (116)	535 (18)	390 (134)	456 (34)	522 (250)	472 (127)	379 (82)	388 (77)	474 (21)
PFOS	0.05	232 (34)	194 (65)	271 (22)	233 (50)	330 (24)	273 (59)	400 (18)	429 (183)	411 (111)	368 (59)	390 (79)	435 (72)
9Cl	0.03	40 (5.9)	23 (8.1)	30 (2.2)	25 (5.7)	36 (7.0)	27 (5.2)	44 (2.0)	52 (21)	55 (25)	41 (10)	46 (12)	61 (3.4)
11Cl	0.04	4.7 (0.1)	4.0 (1.4)	5.1 (1.0)	4.0 (0.9)	6.5 (1.1)	4.1 (1.0)	6.8 (0.3)	6.9 (2.8)	10 (3.9)	6.5 (2.3)	6.3 (1.5)	11 (0.9)
ADONA	0.04	20 (3.6)	17 (7.8)	30 (3.3)	33 (8.5)	52 (0.9)	39 (10)	39 (3.6)	70 (33)	80 (23)	41 (19)	46 (6.7)	78 (4.8)

Table S11. Concentration of PFAS in the control substrate on ash basis [ng g^{-1} ash]. *Only one out of three replicates had levels above the limit of quantification (LOQ). Mean is given as the level in that sample divided on the number of replicates. See Table S17 for decision rule for when to substitute observations below the LOQ.

				Inoculation			Harvest 1			Harvest 2		
	**Day 3**	**Day 6**	**Day 9**	**Sub**	**Bisp**	**No**	**Sub**	**Bisp**	**No**	**Sub**	**Bisp**	**No**
**C3**	34 (20)	10*	40 (9.1)	65 (22)	31 (18)	33 (18)	<LOQ	<LOQ	<LOQ	<LOQ	<LOQ	<LOQ
**C4**	<LOQ	<LOQ	<LOQ	1.9*	<LOQ	<LOQ	<LOQ	<LOQ	<LOQ	<LOQ	<LOQ	<LOQ
**C5**	<LOQ	<LOQ	0.36*	2.7 (1.6)	1.4 (0.55)	2.5 (1.1)	12 (6.2)	1.8 (1.9)	1.6 (0.66)	5.6 (2.1)	2.0 (0.38)	3.6 (0.96)
**C6**	1.6 (1.1)	4.7 (2.4)	3.8 (0.14)	8.9 (2.9)	4.6 (1.7)	6.5 (1.9)	5.1 (1.6)	5.5 (2.7)	3.3 (0.76)	5.0 (0.37)	5.2 (0.36)	7.7 (1.7)
**C7**	1.3 (1.0)	3.1 (2.3)	4.3 (0.25)	5.9 (2.5)	3.2 (2.0)	5.7 (2.1)	3.1 (1.0)	3.5 (2.7)	2.1 (0.73)	3.5 (0.50)	3.5 (0.50)	6.2 (2.2)
**C8**	3.9 (0.55)	4.4 (2.3)	4.7 (0.58)	5.7 (2.5)	4.1 (1.1)	5.0 (0.90)	4.7 (1.4)	5.0 (2.5)	1.9 (0.59)	5.0 (0.72)	4.3 (0.41)	5.4 (1.2)
**C9**	2.7 (0.56)	5.0 (3.1)	3.5 (1.1)	3.5 (2.0)	2.8 (1.1)	3.1 (1.1)	3.0 (1.1)	4.4 (2.5)	1.0 (0.60)	4.2 (1.1)	3.7 (0.71)	3.2 (0.80)
**C10**	3.8 (0.81)	5.7 (2.9)	3.8 (1.6)	4.0 (2.1)	3.5 (1.7)	3.2 (0.57)	3.5 (1.2)	5.1 (2.5)	1.5 (0.80)	5.0 (1.3)	4.7 (1.1)	3.4 (1.0)
**C11**	2.3 (0.10)	3.2 (3.9)	2.8 (0.58)	1.8 (0.99)	2.1 (1.0)	1.5 (0.59)	1.8 (0.52)	3.3 (1.5)	1.1 (0.54)	3.0 (1.2)	2.6 (0.77)	1.9 (0.69)
**C12**	3.1 (1.3)	5.8 (1.8)	2.8 (0.92)	2.1 (1.3)	2.4 (1.2)	1.7 (0.68)	1.6 (0.58)	4.8 (2.9)	1.2 (0.42)	2.7 (1.3)	2.6 (1.1)	1.9 (0.98)
**C13**	3.8 (1.6)	7.8 (1.6)	3.3 (0.57)	2.0 (1.3)	2.6 (1.0)	1.9 (1.2)	1.5 (0.57)	5.3 (3.6)	1.2 (0.38)	2.5 (1.2)	2.6 (1.3)	1.9 (0.76)
**C14**	3.4 (1.4)	7.2 (1.0)	3.2 (0.71)	2.0 (1.4)	2.3 (1.5)	1.7 (0.29)	1.2 (0.51)	5.9 (6.1)	0.93 (0.30)	2.1 (0.95)	2.0 (1.0)	1.9 (0.89)
**PFBS**	<LOQ	1.3 (1.3)	0.27*	1.1 (0.72)	0.70 (0.68)	1.4 (0.47)	1.2 (0.34)	1.3 (0.63)	0.78 (0.11)	0.9 (0.14)	0.9 (0.28)	1.5 (0.73)
**PFHxS**	4.3 (2.0)	3.4 (1.4)	2.9 (1.2)	4.7 (1.9)	3.4 (1.1)	4.4 (0.30)	2.8 (0.96)	3.2 (1.9)	0.39 (0.18)	2.8 (0.94)	2.0 (0.34)	1.5 (0.94)
**PFOS**	65 (51)	14 (18)	18 (13)	74 (48)	43 (33)	34 (18)	2.4 (0.68)	3.0 (1.1)	0.30 (0.20)	3.1 (1.1)	2.2 (0.53)	0.85 (0.4)
**9Cl**	0.35 (0.11)	0.73 (0.20)	<LOQ	<LOQ	<LOQ	<LOQ	0.25 (0.11)	0.28 (0.19)	<LOQ	0.32 (0.17)	0.25 (0.044)	0.041*
**11Cl**	<LOQ	<LOQ	<LOQ	<LOQ	<LOQ	<LOQ	<LOQ	<LOQ	<LOQ	<LOQ	<LOQ	<LOQ
**ADONA**	<LOQ	0.11 (0.20)	<LOQ	0.30 (0.16)	0.23 (0.074)	<LOQ	0.18 (0.058)	0.13*	0.18 (0.068)	0.29 (0.051)	0.29 (0.13)	0.51 (0.19)

Table S12. Concentration of PFAS in the spiked substrate on ash basis [ng g^{-1} ash] with standard deviation in parenthesis.

				Inoculation			Harvest 1			Harvest 2		
	**Day3**	**Day 6**	**Day 9**	**Bisp**	**No**	**Sub**	**Bisp**	**No**	**Sub**	**Bisp**	**No**	**Sub**
**C3**	3304 (2778)	975 (379)	1454 (247)	873 (65)	1993 (1262)	1416 (1206)	867 (521)	661 (326)	314 (252)	448 (279)	1154 (1058)	189 (165)
**C4**	729 (223)	403 (125)	780 (102)	655 (49)	456 (168)	477 (214)	297 (174)	378 (132)	266 (175)	242 (123)	1186 (1117)	179 (146)
**C5**	942 (211)	626 (190)	1297 (101)	1884 (138)	1066 (409)	1262 (704)	831 (388)	844 (168)	656 (185)	522 (235)	1700 (1051)	472 (325)
**C6**	1222 (170)	930 (349)	2058 (157)	3315 (240)	1982 (853)	2193 (1113)	1349 (608)	1445 (196)	981 (93)	797 (287)	1991 (676)	777 (448)
**C7**	1616 (182)	1262 (519)	2510 (191)	3521 (197)	2432 (1047)	2420 (969)	1779 (826)	1882 (282)	1148 (98)	1087 (388)	2509 (753)	1090 (567)
**C8**	1835 (230)	1332 (491)	2414 (104)	2810 (192)	2097 (669)	2024 (710)	2185 (1030)	2259 (557)	1396 (69)	1360 (490)	3234 (1001)	1348 (566)
**C9**	2023 (225)	1399 (543)	2416 (301)	2490 (58)	1977 (463)	1849 (601)	2286 (1103)	2349 (565)	1574 (79)	1567 (570)	3862 (1126)	1576 (624)
**C10**	2395 (223)	1633 (533)	2638 (357)	2621 (100)	2185 (416)	2027 (690)	2317 (1071)	2334 (490)	1727 (108)	1697 (588)	3450 (1109)	1653 (532)
**C11**	1534 (181)	1139 (401)	1733 (268)	1667 (76)	1420 (274)	1346 (440)	1424 (670)	1462 (392)	1102 (57)	1014 (358)	2217 (676)	1080 (351)
**C12**	1868 (224)	1332 (485)	2025 (400)	1900 (142)	1654 (253)	1503 (494)	1276 (543)	1505 (381)	1115 (57)	995 (361)	2096 (491)	1055 (280)
**C13**	2272 (180)	1629 (619)	2487 (611)	2138 (171)	1873 (228)	1673 (528)	1161 (507)	1644 (363)	1120 (38)	921 (356)	2222 (584)	1069 (281)
**C14**	2104 (145)	1540 (646)	2366 (621)	1862 (161)	1812 (289)	1445 (447)	799 (328)	1469 (483)	894 (32)	676 (261)	1725 (326)	815 (184)
**PFBS**	718 (138)	514 (126)	765 (139)	890 (52)	579 (120)	671 (276)	544 (242)	482 (96)	469 (90)	309 (114)	928 (662)	350 (170)
**PFHxS**	2108 (256)	1616 (574)	2912 (193)	3302 (26)	2264 (876)	2308 (939)	2020 (1015)	1762 (409)	1585 (111)	1180 (458)	2601 (982)	1343 (358)
**PFOS**	1763 (170)	1251 (421)	1971 (311)	2041 (200)	1582 (416)	1527 (446)	1664 (760)	1536 (360)	1394 (81)	1195 (486)	2326 (611)	1303 (271)
**9Cl**	301 (33)	151 (56)	219 (35)	225 (49)	157 (37)	167 (49)	200 (88)	204 (86)	153 (8.1)	141 (63)	336 (123)	146 (44)
**11Cl**	36 (2.2)	26 (11)	37 (9.7)	40 (7.2)	24 (6.8)	27 (7.9)	27 (12)	36 (13)	24 (1.4)	19 (8.7)	58 (20)	23 (10)
**ADONA**	148 (24)	107 (41)	219 (7.5)	320 (7.3)	224 (70)	214 (72)	274 (137)	299 (76)	137 (10)	141 (52)	428 (155)	146 (77)

Table S13. Limit of quantification (LOQ) and mean PFAS concentration in the mushroom hats on fresh weight basis (ng g^{-1} fw), with standard deviation in parenthesis. When the average concentration of a PFAS is below the LOQ, it is because one or two of the replicates had a concentration below the LOQ which was replaced by LOQ/sqrt(2). * There was uptake in only one replicate, the concentration of that replicate divided by the number of replicates are given. See Table S17 for decision rule for when to substitute observations below the LOQ.

		Control				Spiked			
		Harvest 1		Harvest 2		Harvest 1		Harvest 2	
	LOQ	Sub	Bisp	Sub	Bisp	Sub	Bisp	Sub	Bisp
**PFPrA**	**1.3**	<1.3	<1.3	0.58*	<1.3	0.55*	1.0*	2.2 (0.93)	3.4 (2.1)
**PFBA**	**0.71**	<0.71	<0.71	<0.71	<0.71	<0.71	0.31*	<0.71	1.1 (0.50)
**PFPeA**	**0.05**	<0.05	<0.05	<0.05	<0.05	<0.05	0.06*	<0.05	0.43 (0.49)
**PFHxA**	**0.03**	<0.03	0.03 (0.01)	0.016*	<0.03	<0.03	0.14 (0.15)	0.13 (0.01)	0.24 (0.11)
**PFHpA**	**0.03**	<0.03	<0.03	0.011*	<0.03	<0.03	0.11 (0.11)	0.08 (0.00)	0.06 (0.05)
**PFOA**	**0.04**	0.05 (0.01)	0.05 (0.01)	0.05 (0.01)	<0.04	0.059 (0.01)	0.12 (0.08)	0.08 (0.00)	0.06 (0.01)
**PFNA**	**0.04**	<0.04	<0.04	<0.04	<0.04	<0.04	0.10 (0.08)	<0.04	0.05 (0.03)
**PFDA**	**0.03**	<0.03	<0.03	<0.03	<0.03	0.012*	0.09 (0.09)	0.06 (0.05)	0.07 (0.07)
**PFUnDA**	**0.05**	<0.05	<0.05	<0.05	<0.05	<0.05	0.08 (0.07)	0.06 (0.04)	0.07 (0.05)
**PFDoDA**	**0.17**	<0.17	<0.17	<0.17	<0.17	<0.17	<0.17	<0.17	<0.17
**PFTriDA**	**0.05**	<0.05	<0.05	<0.05	<0.05	<0.05	0.13 (0.13)	0.06 (0.04)	0.06 (0.04)
**PFTeDA**	**1.06**	<1.06	<1.06	<1.06	<1.06	<1.06	<1.06	<1.06	<1.06
**PFBS**	**0.02**	<0.02	<0.02	0.012*	<0.02	<0.02	0.10 (0.11)	0.08 (0.00)	0.15 (0.04)
**PFHxS**	**0.03**	<0.03	<0.03	0.013*	0.01*	0.011*	0.12 (0.11)	0.08 (0.07)	0.03 (0.01)
**PFOS**	**0.03**	<0.03	<0.03	<0.03	<0.03	<0.03	0.05 (0.04)	0.05 (0.04)	0.04 (0.03)
**9Cl**	**0.05**	<0.05	<0.05	<0.05	<0.05	<0.05	<0.05	<0.05	<0.05
**11Cl**	**0.06**	<0.06	<0.06	<0.06	<0.06	<0.06	<0.06	<0.06	<0.06
**ADONA**	**0.06**	<0.06	<0.06	<0.06	<0.06	<0.06	<0.06	<0.06	<0.06

Table S14. Mean PFAS concentration in the mushroom hats on a dry weight basis, with standard deviation given in parenthesis. * There was uptake in only one replicate, the concentration of that replicate divided by the number of replicates are given. See Table S17 for decision rule for when to substitute observations below the limit of quantification (LOQ).

	Control				Spiked			
	Harvest 1		Harvest 2		Harvest 1		Harvest 2	
	Sub	Bisp	Sub	Bisp	Sub	Bisp	Sub	Bisp
**PFPrA**	<LOQ	<LOQ	4.2*	<LOQ	4.2*	7.9*	14 (6.5)	28 (21)
**PFBA**	<LOQ	<LOQ	<LOQ	<LOQ	<LOQ	2.4*	<LOQ	9.0 (5.5)
**PFPeA**	<LOQ	<LOQ	<LOQ	<LOQ	<LOQ	0.46*	<LOQ	3.7 (4.5)
**PFHxA**	<LOQ	0.27 (0.11)	0.14*	<LOQ	<LOQ	1.2 (1.1)	0.78 (0.12)	2.0 (1.2)
**PFHpA**	<LOQ	<LOQ	0.10*	<LOQ	<LOQ	0.91 (0.79)	0.50 (0.03)	0.47 (0.47)
**PFOA**	0.33 (0.09)	0.40 (0.11)	0.35 (0.16)	<LOQ	0.45 (0.11)	1.0 (0.55)	0.48 (0.04)	0.50 (0.15)
**PFNA**	<LOQ	<LOQ	<LOQ	<LOQ	<LOQ	0.80 (0.62)	<LOQ	0.43 (0.33)
**PFDA**	<LOQ	<LOQ	<LOQ	<LOQ	0.09*	0.79 (0.64)	0.34 (0.29)	0.60 (0.64)
**PFUnDA**	<LOQ	<LOQ	<LOQ	<LOQ	<LOQ	0.71 (0.48)	0.38 (0.21)	0.59 (0.49)
**PFDoDA**	<LOQ	<LOQ	<LOQ	<LOQ	<LOQ	<LOQ	<LOQ	<LOQ
**PFTriDA**	<LOQ	<LOQ	<LOQ	<LOQ	<LOQ	1.1 (0.94)	0.38 (0.21)	0.52 (0.39)
**PFTeDA**	<LOQ	<LOQ	<LOQ	<LOQ	<LOQ	<LOQ	<LOQ	<LOQ
**PFBS**	<LOQ	<LOQ	0.10*	<LOQ	<LOQ	0.77 (0.80)	0.46 (0.03)	1.2 (0.53)
**PFHxS**	<LOQ	<LOQ	0.11*	0.09*	0.09*	1.1 (0.83)	0.50 (0.38)	0.23 (0.13)
**PFOS**	<LOQ	<LOQ	<LOQ	<LOQ	<LOQ	0.46 (0.28)	0.31 (0.25)	0.35 (0.30).
**9Cl**	<LOQ	<LOQ	<LOQ	<LOQ	<LOQ	<LOQ	<LOQ	<LOQ
**11Cl**	<LOQ	<LOQ	<LOQ	<LOQ	<LOQ	<LOQ	<LOQ	<LOQ
**ADONA**	<LOQ	<LOQ	<LOQ	<LOQ	<LOQ	<LOQ	<LOQ	<LOQ

Table S15. Log 10 Bioaccumulation factors, with standard deviation in parenthesis. *There was mushroom uptake in only one replicate. The BAF given in the table is the BAF of that replicate, not divided by the number of replicates (unlike what has been done for the concentration data). C12 (PFDoDA), C14 (PFTeDA), ADONA, and F-53B compounds are not included in the table as there was no detectable uptake of these compounds in any of the mushroom samples.

	Control				Spiked			
	Harvest 1		Harvest 2		Harvest 1		Harvest 2	
	Sub	Bisp	Sub	Bisp	Sub	Bisp	Sub	Bisp
C3					-1.14*	-0.98*	-0.33 (0.27)	-0.85 (0.56)
C4						-1.23*		-1.05 (0.36)
C5						-2.31*		-1.99 (0.75)
C6		-0.75 (0.35)	-0.36*			-2.64 (0.63)	-2.29 (0.07)	-2.20 (0.26)
C7			-0.42*			-2.83 (0.57)	-2.65 (0.01)	-3.05 (0.52)
C8	-0.58 (0.02)	-0.52 (0.28)	-0.61 (0.22)		-2.96 (0.14)	-2.75 (0.27)	-2.80 (0.02)	-2.99 (0.16)
C9						-2.94 (0.45)		-3.20 (0.41)
C10					-3.25*	-2.99 (0.52)	-3.16 (0.47)	-3.19 (0.61)
C11						-2.75 (0.34)	-2.87 (0.31)	-2.87 (0.37)
C13						-2.55 (0.47)	-2.89 (0.31)	-2.84 (0.24)
PFBS			0.22*			-2.39 (0.34)	-2.21 (0.06)	-1.98 (0.18)
PFHxS			-0.30*	-0.22*	-3.20*	-2.81 (0.59)	-2.90 (0.42)	-3.29 (0.31)
PFOS						-3.01 (0.39)	-3.12 (0.44)	-3.18 (0.44)

Table S16. "Worst-case" – bioaccumulation factors (not log-transformed). When there are empty cells, it means there was uptake in all the replicates of the treatment. For some compounds, a value is given both in Table S6 and in Table S7. This happens when there is uptake above the LOQ in some of the replicates but not all. Then the values above the LOQ, and possible imputations, are used to calculate the real BAFs in Table S6, while the LOQ-values are used to calculate the worst-case BAFs.

	**Control**				**Spiked**			
	**Harvest 1**		**Harvest 2**		**Harvest 1**		**Harvest 2**	
	**Sub**	**Bisp**	**Sub**	**Bisp**	**Sub**	**Bisp**	**Sub**	**Bisp**
PFPrA	3.8	4.8	2.9	5.1	0.14	0.13		
PFBA	4.7	6.0	4.8	6.3	0.09	0.10	0.16	
PFPeA	0.14	1.6	0.28	0.81	<0.01	<0.01	<0.01	
PFHxA	0.17	0.07	0.12	0.18	<0.01	<0.01		
PFHpA	0.29	0.40	0.18	0.27	<0.01	<0.01		<0.01
PFOA	0.10	0.08	0.04	0.29				
PFNA	0.41	0.38	0.28	0.35	<0.01	<0.01	<0.01	<0.01
PFDA	0.26	0.23	0.17	0.21	<0.01	<0.01	<0.01	<0.01
PFUnDA	0.82	0.62	0.51	0.66	<0.01	<0.01	<0.01	<0.01
PFDoDA	3.1	1.9	2.0	2.4	<0.01	<0.01	<0.01	<0.01
PFTriDA	0.99	0.58	0.65	0.77	<0.01	<0.01	<0.01	<0.01
PFTeDA	27	14	17	20	0.03	0.05	0.03	0.04
PFBS	0.46	0.58	0.46	0.85	<0.01			
PFHxS	0.31	0.41	0.25	0.39	<0.01	<0.01		<0.01
PFOS	0.35	0.36	0.30	0.45	<0.01	<0.01	<0.01	<0.01
9Cl	7.2	10	5.2	6.6	<0.01	0.01	<0.01	<0.01
11Cl	11	13	11	14	0.07	0.09	0.07	0.07
ADONA	9.4	11	5.8	8.8	0.01	0.01	0.01	<0.01

Spiking procedure

The PFAS spiking solution was poured into the liquid digestate and mixed with a hand-held cement mixer (Elektromix SY-HM-140; Yongkang Well-King Industry and Trade Co. Ltd., China), prior to mixing with straw, chalk, gypsum, and hot compost. The solution container was rinsed three times with 10 ml methanol which was poured into the digestate. The same procedure was done in the control treatment, where the corresponding amount of methanol and water was added. The control batch was always handled first to limit the risk of cross contamination.

Preparation of mushroom substrate

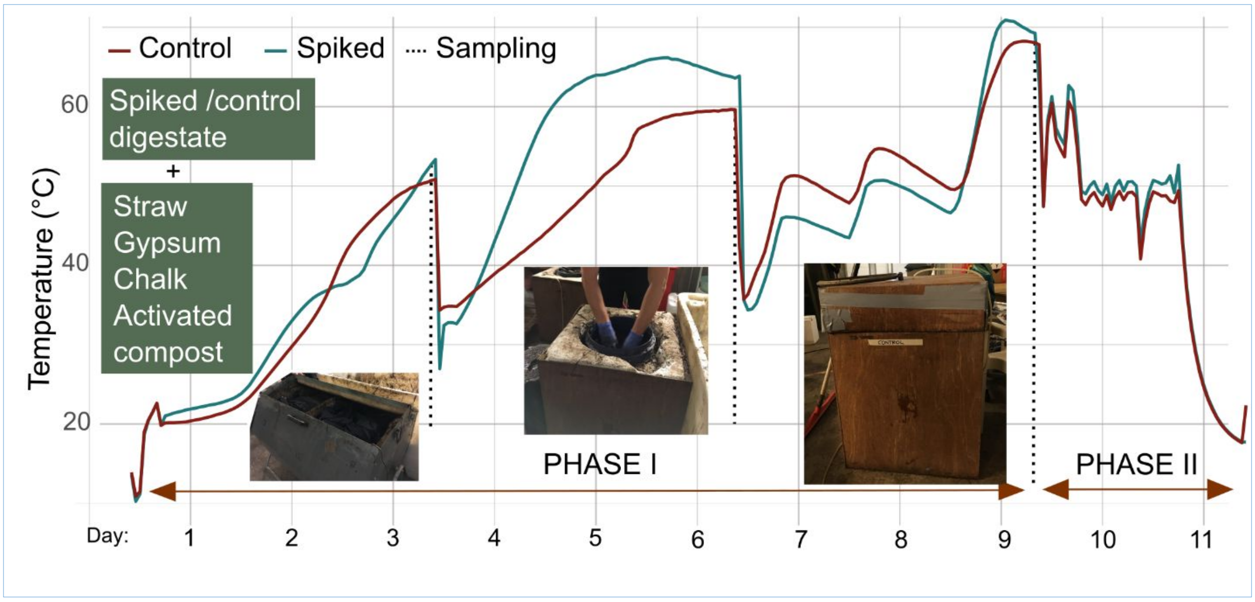

Figure S1. Preparation of the mushroom substrate. Phase I: Composting, Phase II: Pasteurisation. The blue and red lines show the temperature (°C) development in the spiked and control batches, respectively. The night between day 7 and 8 there was a power break which prolonged the composting process by one day. At day 11 (inoculation day), the substrate was inoculated with fungi and spiked with pharmaceuticals.

Phase I – Composting. The ingredients were mixed well before being composted in two separated composting drums (one for each batch – control and spiked, JK270; Joraform; Mjölby. Sweden) for three days until there was a volume loss due to heat generation. Thereafter the substrate was mixed with a hay fork and moved to two 60 L plastic containers (Peguform, Bötzingen, Germany) with a sealed lid and controlled air flow (to obtain similar conditions as in commercial bulk systems; 70-80°C and 6-9% O_2 v/v). The O_2 levels was measured three times a day with a portable gas analyser (GA5000, GeoTech, QED Environonmental Systems Ltd., Coventry, UK). The containers were built into a plywood box and polystyrene was used as isolation between the container and plywood box. A further description can be

found in Stoknes et al. (2013). The substrate was turned twice (by use of the hay fork) at an interval of 3 days, giving a total of 9 days for Phase I. The temperature development in the spiked and control batches are found in Figure S1 during Phase I and II along with pictures from the preparation process.

Phase II – Pasteurisation. The mushroom substrate was moved into a miniature Phase II tunnel, where temperature was slowly increased to 56-60°C over 24 hours and then maintained for 6 hours of pasteurisation. A rebuild 400 L freezer box was used as a Phase II tunnel. The freezer had stainless steel sheet dividers which allowed both batches to be treated simultaneously (see Stoknes et al. (2013) for details). During conditioning the temperature was decreased to 55°C for another 6 hours. Then the temperature was kept at 46-50°C until ammonia (NH_3 gas) had dissipated below 10 ppm. The NH_3 gas level was measured with Dräger accuro Pump with gas detection tubes of the type Ammonia 2/1 2-30 ppm (67 33 231; Dräger Inc, Huston, TX, USA). About 10 kg material was lost during composting, and at the end of Phase II there was 29.7 kg of control mushroom substrate, and 30.8 kg of spiked mushroom substrate.

Mushroom cultivation – additional information.

Casing material: The casing material was dark peat (from Holmen transport) mixed with $Ca(OH)_2$ to increase the pH of the casing material up to 7 – 7.5 and with gypsum for structure maintenance (120 g gypsum / 40 l peat). Casing is crucial for the development of fruiting bodies (i.e.. mushroom hats), as it holds enough water for basidiocarps to develop properly. The casing layer was sprinkled for the first few days with distilled water. The opened bags were moved to a cultivation chamber holding 25-30°C, where the air humidity was kept at 70-75%. The chamber received LED-light with colour temperature of 6000 K.

Harvesting. The mushroom fruiting bodies were picked/harvested from the individual growing bags as they obtained maturity. The maturity in mushrooms can be described as when the cap of the mushroom is starting to open, however the veil under the mushroom cap is still intact and attached to the cap edges. The gills are pink and the spores has not yet been released. All mushrooms in a single bag were harvested at once. Each bag had 1-3 harvests/flushes, of which the first was harvested 24 days after the casing was applied (83 days after inoculation) and the last was harvested 12 weeks after the casing day. For those units where the mushrooms had three flushes. the third was not analysed for PFAS.

Sampling procedure for the mushroom substrate

During phase I and II, small amounts of substrate were collected from a variety of spots in the batch and combined to a composite sample. The composite sample was cut into small pieces by a scissor and mixed well before the substrate was weighed into polypropylene sample tubes for the various analysis. When the casing was applied, the composite sample was made by taking three subsamples from each

bag: one from the top, one from the bottom, and one from the middle by making a small hole in the bag and taking out the substrate with an awl. Dry matter, ash, and pH were determined immediately. Samples for all other analysis were kept at -18°C until analysis.

Temperature logging

The temperature was monitored by loggers from Onset HOBO S-WCA-M003 (Bourne. MA. USA). During Phase I and II, three loggers were placed in each compost, one in the middle and two towards the sides. During mushroom cultivation there was one logger in the middle of each bag.

Substrate extraction procedure

Substrate samples were homogenized and then oven dried at 70˚C for 12 hrs. A 1 g (dry weight) sub-sample was then weighed into a polypropylene centrifuge tube and fortified with isotopically labelled internal standards (complete list provided in Table S4). Methanol (5 ml) was added, and the samples were shaken vigorously for 5 min, sonicated for 15 minutes, and then centrifuged (3000 rpm, 10 min). The resulting supernatant was transferred to a 13 ml PP tube, and after repeating the extraction once the supernatants were combined and concentrated under a stream of nitrogen to approximately 1 ml. The extract was transferred to a 1.7 ml Eppendorf centrifuge tube containing 25 mg ENVI-Carb and 50 µl glacial acetic acid, vortexed, and centrifuged (3000 rpm, 10 min). A portion (300 µl) of the extract was transferred to a microvial and fortified with 25 µl of a recovery standard solution (Table S4) and 300 µl of 4 mM NH_4OAc in water prior to instrumental analysis.

Mushroom extraction procedure

Mushroom samples were extracted by placing 1 g (wet weight) into a centrifuge tube together with 8 stainless steal beads (4.8 mm diameter) and 5 ml acetonitrile. The samples were then placed in a bead blender (SPEX SamplePrep 1600 MiniG) for 5 min at 1500 rpm, then centrifuged (3000 rpm, 10 min). The supernatant was removed to a 13 ml PP tube, and the extraction was repeated; thereafter the supernatants were combined and concentrated under a stream of nitrogen to approximately 1 ml. The extract was transferred to a 1.7 ml Eppendorf centrifuge tube containing 25 mg ENVI-Carb and 50 µl glacial acetic acid, vortexed, and centrifuged (3000 rpm, 10 min). A portion of the extract (300 µl) was transferred to a microvial and fortified with 25 µl of a recovery standard solution (Table S4) and 300 µl of 4 mM NH_4OAc in water prior to instrumental analysis.

Instrumental analysis

Mushroom and substrate extracts were analysed using two different methods. PFPrA, PFBA, and PFPeA were analysed with a Dionex Ultimate 3000 liquid chromatograph equipped with a Thermo Scientific Acclaim Trinity P1 column (3 µm, 2.1 × 100mm) and coupled to a Q Exactive HF Orbitrap (Thermo Scientific). The instrument was operated in negative electrospray ionization, full scan-data dependent MS2 mode, using a list of parent ions for PFPrA, PFBA, and PFPeA as an inclusion list. Finally, C6-C14 PFCAs, C4, C6, and C8 PFSAs, ADONA, 9Cl-PF3ONS, and 11Cl-PF3OUdS were determined using an Acquity UPLC equipped with a Waters BEH C18 guard column (5 × 2.1 mm, 1.7 µm) and a Waters BEH C18 analytical column (50 × 2.1 mm, 1.7 µm). The instrument was operated in negative electrospray ionization, multiple-reaction-monitoring mode. Quantification was carried out using an internal standard or isotope dilution approach, with a 5 to 8 point linear calibration curve with 1/x weighting, excluding the origin (see tables S2-S4 for PFAS and their corresponding internal standards). Correlation coefficients (r^2) were >0.99 for all targets with the exception of PFTrDA (>0.85), PFTeDA (>0.95), PFDS (>0.95), and 11Cl-PF3OUdS (>0.98), which were lower, possibly due to use of non-exactly matched isotopically labelled internal standards. Nevertheless, since accuracy of QC samples was reasonable for these targets, the curves were deemed sufficient for quantification of samples. Details of all instrumental methods, including LC gradients are provided in Tables S2, S3, and S4.

Quality control

To account for laboratory background contamination, procedural blanks (no matrix) were processed together with all samples. Among the remaining ultra-short chain PFAS analysed by LC-Orbitrap MS, blanks (n=2 for mushrooms and n=4 for substrates) displayed detectable levels of PFBA; consequently, LODs for both substrates and mushrooms were defined as the average concentration in the blank plus 3 time the standard deviation of the blanks. PFPrA and PFPeA were not observable in blanks and therefore LODs were based on the concentration of the lowest calibration point. Finally, for the remaining targets, LODs were based on the higher of either the lowest point on the calibration curve or the average concentration in the blanks plus 3 time the standard deviation of the blanks (n=3 for substrates and n=2 for mushrooms).

To assess accuracy and precision, replicate spike/recovery experiments were performed using mushrooms purchased from the grocery store (Agaricus bisporus; n=6 fortified with 39 – 180 ng of individual PFAS; n=3 unfortified, Table S5) as well as in-house pooled fish muscle (chosen due to low background PFAA contamination; n=6 fortified with 2.5 - 200 ng of individual PFAS; n=3 unfortified, Table S6). Percent recoveries, calculated by comparing the measured concentrations to expected concentrations in the spiked samples (after accounting for background levels) ranged from 88 – 119%

(standard deviation: 1 – 25%) in mushrooms. In fish muscle, recoveries were 101 – 129% (standard deviation 5 – 20%) for most PFAS, with the exception of PFBS which showed higher recoveries than expected (168 ± 20%, respectively) and 11Cl-PF3OUdS, which displayed lower recoveries than expected (51±8%), which was attributed to the use of non-exactly matched isotopically labelled internal standards for these substances. Overall, however, accuracy and precision were deemed suitable for the present work. In addition, samples of NIST 2781 (domestic sludge) were extracted and compared to measurements reported elsewhere (Table S7). In general, concentrations were either within the range of those reported previously or slightly lower. Most notably, concentrations of PFBA (determined using the Trinity P1 column) were considerably lower (2.33 ± 0.19 ng/g compared to 3.34 – 35.9 ng/g reported previously using C18 columns). Recently a PFBA interference, 3 oxo-dodecanoic acid, was identified in biological samples (Bangma et al.. 2021), and we speculate that this substance may also exist in SRM 278, but was removed using the Trinity P1 column, resulting in lower concentrations than reported elsewhere. Overall, these data indicate reasonable accuracy and precision of the method.

Substitution of values below the limit of quantification

Values below LOQ were substituted with LOQ/sqrt(2) as described elsewhere (Nyberg et al., 2018), in certain cases. About 80% of the observations of PFAS in the mushroom hats were below the LOQ. Replacing all <LOQ values by e.g., LOQ/sqrt(2) gives the following problems: (i) The mushroom uptake would seem higher for those compounds with higher LOQs, even when there was no detectable uptake in any of the mushroom hats. (ii) The analysis of PFAS in mushrooms were done on a wet weight basis before the concentrations was recalculated into dry weight basis. Even in cases where all replicates were <LOQ, the mushroom concentration would have a standard deviation due to the varying dry matter content of the mushrooms.

Therefore, it was decided to replace the <LOQ observations by the LOQ/square root of 2 only in certain cases, as seen in the following overview. The number of replicates for one treatment (combination of species and control/spiked) at one sampling time was between 2 and 4. The rules were applied for the mushroom substrate as well, where about 20% of the observations were <LOQ.

Table S17. Decision rule for imputation of observations below the limit of quantification (LOQ)

Number of replicates >LOQ	Replace <LOQ?	Mean given as
None	No	<LOQ
One (in cases with 3 or 4 replicates)	No	The concentration in the >LOQ sample divided by the number of replicates (labelled by a star)
At least 50%<br>(1 when there are 2 replicates,<br>2 when there is 3 or 4)	Yes, by<br>LOQ / sqrt(2)	The mean of all replicates, including imputations.

```
Linear mixed model fit by REML. t-tests use Satterthwaite's method ['lmerModLmerTest']
Formula: C8 ~ Treatment + Time + Species + (1 | Unit)
   Data: C8

REML criterion at convergence: 6.5

Scaled residuals:
    Min      1Q  Median      3Q     Max
-1.1261 -0.3600 -0.0645  0.4167  1.2407

Random effects:
 Groups   Name        Variance Std.Dev.
 Unit     (Intercept) 0.06816  0.2611
 Residual             0.01626  0.1275
Number of obs: 22, groups:  Unit, 14

Fixed effects:
                Estimate Std. Error       df t value Pr(>|t|)
(Intercept)      0.27047    0.12560 10.27406   2.153   0.0560 .
TreatmentSpiked  0.32465    0.15233 10.04458   2.131   0.0588 .
TimeH2          -0.03765    0.06290  6.55778  -0.598   0.5696
SpeciesBisp      0.22053    0.15195 10.40008   1.451   0.1762
---
Signif. codes:  0 '***' 0.001 '**' 0.01 '*' 0.05 '.' 0.1 ' ' 1

Correlation of Fixed Effects:
            (Intr) TrtmnS TimeH2
TretmntSpkd -0.502
TimeH2      -0.199 -0.037
SpeciesBisp -0.590 -0.029  0.117
```

Figure S2. Result of linear mixed model with treatment (spiked/control), harvest time, and mushroom species as fixed effects, experimental units as random effects, and concentration of PFOA in the mushrooms as the dependent variable.

```
Call:
lm(formula = LogBAF ~ CF2, data = pFCA_C3toC7_Spiked)

Residuals:
     Min       1Q   Median       3Q      Max 
-1.00070 -0.34628  0.09527  0.29432  0.62029 

Coefficients:
            Estimate Std. Error t value Pr(>|t|)    
(Intercept)  0.85763    0.29827   2.875  0.00833 ** 
CF2         -0.53839    0.05474  -9.836 6.79e-10 ***
---
Signif. codes:  0 ‘***’ 0.001 ‘**’ 0.01 ‘*’ 0.05 ‘.’ 0.1 ‘ ’ 1

Residual standard error: 0.4262 on 24 degrees of freedom
  (24 observations deleted due to missingness)
Multiple R-squared:  0.8012,	Adjusted R-squared:  0.7929 
F-statistic: 96.74 on 1 and 24 DF,  p-value: 6.787e-10
```

Figure S3. Result of linear regression on the log10 BAFs of the PFCAs C3 through C7. Non-detects are not included in the regression. CF2 equals the number of carbons in the compounds.

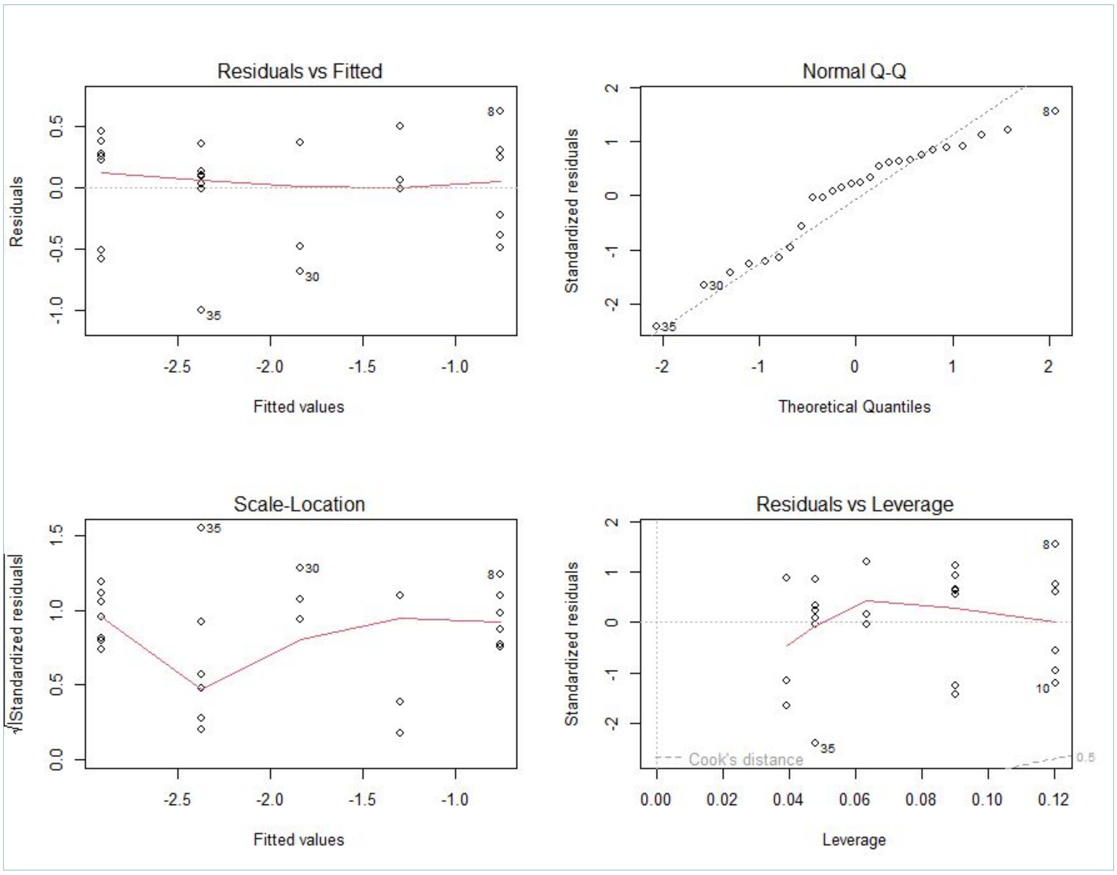

Figure S4. Diagnostic plot for the linear regression of the log BAF as a function of carbon chain length, for the PFCAs C3 to C7.

```
Call:
lm(formula = LogBAF ~ CF2, data = PFSA_Spiked)

Residuals:
    Min      1Q  Median      3Q     Max 
-0.7328 -0.2880  0.1263  0.3127  0.5321 

Coefficients:
            Estimate Std. Error t value Pr(>|t|)    
(Intercept) -1.47734    0.32852  -4.497 0.000220 ***
CF2         -0.21733    0.05291  -4.107 0.000548 ***
---
Signif. codes:  0 '***' 0.001 '**' 0.01 '*' 0.05 '.' 0.1 ' ' 1

Residual standard error: 0.396 on 20 degrees of freedom
  (8 observations deleted due to missingness)
Multiple R-squared:  0.4575,	Adjusted R-squared:  0.4304 
F-statistic: 16.87 on 1 and 20 DF,  p-value: 0.0005476
```

Figure S5. Result of linear regression on the log10 BAFs of the PFSAs PFBS, PFHxS, and PFOS. Non-detects are not included in the regression. CF2 equals the number of carbons in the compounds.

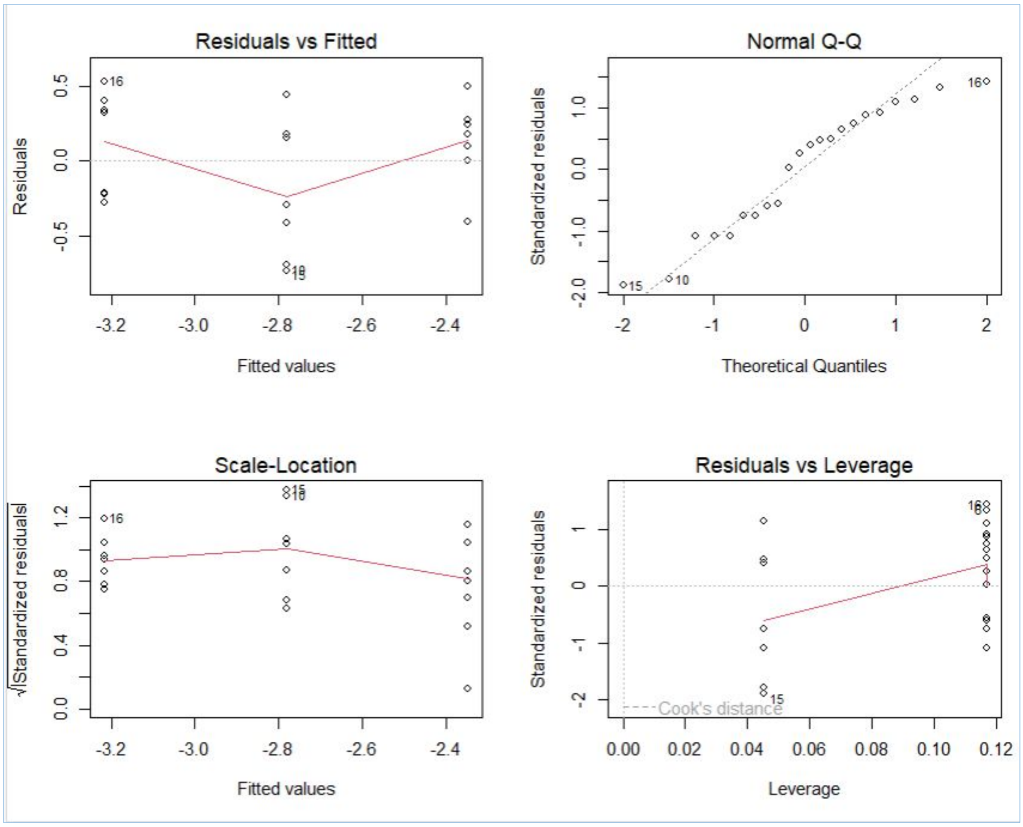

Figure S6. Diagnostic plots for linear regression of the log BAF as a function of carbon chain length, for the PFSAs PFBS, PFHxS, and PFOS.

References

Bangma, J.T., Reiner, J., Fry, R.C., Manuck, T., McCord, J., Strynar, M.J., 2021. Identification of an Analytical Method Interference for Perfluorobutanoic Acid in Biological Samples. Environ. Sci. Technol. Lett. 8, 1085–1090.

Munoz, G., Michaud, A.M., Liu, M., Vo Duy, S., Montenach, D., Resseguier, C., Watteau, F., Sappin-Didier, V., Feder, F., Morvan, T., Houot, S., Desrosiers, M., Liu, J., Sauvé, S., 2021. Target and Nontarget Screening of PFAS in Biosolids, Composts, and Other Organic Waste Products for Land Application in France. Environ. Sci. Technol. acs.est.1c03697.

Nyberg, E., Awad, R., Bignert, A., Ek, C., Sallsten, G., Benskin, J.P., 2018. Inter-individual, inter-city, and temporal trends of per- and polyfluoroalkyl substances in human milk from Swedish mothers between 1972 and 2016. Environ. Sci. Process. Impacts 20, 1136–1147.

Reiner, J.L., Blaine, A.C., Higgins, C.P., Huset, C., Jenkins, T.M., Kwadijk, C.J.A.F., Lange, C.C., Muir, D.C.G., Reagen, W.K., Rich, C., Small, J.M., Strynar, M.J., Washington, J.W., Yoo, H., Keller, J.M., 2015. Polyfluorinated substances in abiotic standard reference materials. Anal. Bioanal. Chem. 407, 2975–2983.

Stoknes, K., Beyer, D.M., Norgaard, E., 2013. Anaerobically digested food waste in compost for Agaricus bisporus and Agaricus subrufescens and its effect on mushroom productivity. J. Sci. Food Agric. 93, 2188–2200.
